# The Recent Progress of MEMS/NEMS Resonators

**DOI:** 10.3390/mi12060724

**Published:** 2021-06-19

**Authors:** Lei Wei, Xuebao Kuai, Yidi Bao, Jiangtao Wei, Liangliang Yang, Peishuai Song, Mingliang Zhang, Fuhua Yang, Xiaodong Wang

**Affiliations:** 1 Engineering Research Center for Semiconductor Integrated Technology, Institute of Semiconductors, Chinese Academy of Sciences, Beijing 100083, China; weilei@semi.ac.cn (L.W.); kuaixuebao@semi.ac.cn (X.K.); baoyidi@semi.ac.cn (Y.B.); weijt@semi.ac.cn (J.W.); yangliangliang@semi.ac.cn (L.Y.); pssong@semi.ac.cn (P.S.); zhangml@semi.ac.cn (M.Z.); fhyang@semi.ac.cn (F.Y.); 2 The School of Microelectronics & Center of Materials Science and Optoelectronics Engineering, University of Chinese Academy of Sciences, Beijing 100049, China; 3 School of Microelectronics, University of Science and Technology of China, Hefei 230026, China; 4 Beijing Academy of Quantum Information Science, Beijing 100193, China; 5 Beijing Engineering Research Center of Semiconductor Micro-Nano Integrated Technology, Beijing 100083, China

**Keywords:** MEMS/NEMS, single/double-clamped resonators, hemispherical shell resonators, ring/microdisk resonators, SAW/BAW resonators

## Abstract

MEMS/NEMS resonators are widely studied in biological detection, physical sensing, and quantum coupling. This paper reviews the latest research progress of MEMS/NEMS resonators with different structures. The resonance performance, new test method, and manufacturing process of single or double-clamped resonators, and their applications in mass sensing, micromechanical thermal analysis, quantum detection, and oscillators are introduced in detail. The material properties, resonance mode, and application in different fields such as gyroscope of the hemispherical structure, microdisk structure, drum resonator are reviewed. Furthermore, the working principles and sensing methods of the surface acoustic wave and bulk acoustic wave resonators and their new applications such as humidity sensing and fast spin control are discussed. The structure and resonance performance of tuning forks are summarized. This article aims to classify resonators according to different structures and summarize the working principles, resonance performance, and applications.

## 1. Introduction

Microelectromechanical systems (MEMS) [1] and nanoelectromechanical systems (NEMS) resonators can be used as biosensing [2], mass sensing [3], and core vibration elements in circuits [4]. MEMS devices realize compact and power-efficient sensing applications through the transduction of physical signals. MEMS resonator materials include Si materials compatible with MEMS process, carbon nanotubes(CNTs) [5,6], graphene sheets(GSs) [7,8], nanowires [9], SiC. Two-dimensional materials, including superconducting ${\mathrm{NbSe}}_{2},$ semiconducting ${\mathrm{MoS}}_{2}$ [10] are often used as NEMS resonators [10]. Different materials can be selected according to Ashby’s approach to be applied to MEMS resonators [11]. The resonance range of MEMS sensors can be from a few hertz [12] to GHz according to the application scenario, such as gravimeter can be as low as 2.3 Hz [12]. The resonant structure is also one of the important features of the resonators. Various resonant structures, such as single or double-clamped beams, hemispherical shells [13], microdisks [14], and forks [15] have different applications and performances.

Single-clamped resonators mainly refer to the cantilevers. They can be used as biosensors, which cause a change in mass or stiffness due to surface biochemical effects, resulting in a change in resonance frequency [2]. Since the axial stress of the single-clamped cantilever is released, the single-clamped cantilever of the same size and structure usually has a higher frequency and quality factor than the double-clamped structure when there is only a compressive axial stress state [16]. However, the existence of tensile stress will make the frequency of double-clamped beams much higher than that of cantilevers of the same size. Cantilever sensors [17] can measure the mass of cells or biomolecules, nucleic acids, pH [17], pressure [18], and the concentration of cell suspensions [19]. In the quantum field, it is possible to study the coupling of superconducting transmission line resonator with the artificial atom [20] or the coupling of Bose–Einstein condensed atoms with a nanoscale cantilever [21].

The hemispherical shell resonators are often used as gyroscopes to achieve inertial navigation. Due to the three-dimensional structure, the symmetry is much improved, and the quality factor of more than 10 million can be achieved [22]. Microdisks, drums, and ring resonators can be used for octave frequency tuning [23], mass sensing [24], and gyroscopes [25]. The microdisk resonator uses a pedestal to support the microdisk to suspend. The microdisk and the pedestal can be manufactured by etching using the same material, or microdisks of different materials can be grown on the pedestal. Drum resonators are mostly membrane structures, such as a single-layer graphene membrane with low mass and low rigidity [26] and silicon nitride membranes with low stress [23]. The ring structure resonator can be used as gyroscopes, supported by a central anchor, and equidistant rings are nested with each other by spokes, and different resonance modes appear under the excitation of electrodes [27]. Surface Acoustic Wave (SAW)/Bulk Acoustic Wave (BAW) sensors are made of piezoelectric materials, and acoustic waves propagate on the surface and inside of the material respectively. SAW sensors have the advantages of quasi-digital output, small size, high sensitivity [28], and high test accuracy. BAW devices can be used as sensors to measure very small masses [29]. The architecture of BAW devices includes Quartz Crystal Microbalances (QCMs), Thickness Shear Mode (TSM), Quartz Crystal Resonator (QCR), Film Bulk Acoustic Resonator (FBAR), or Thin-Film Bulk Acoustic Resonator (TFBAR) devices [30]. A new method using preforms to produce fibers MEMS by a thermal drawing process to realize the electromechanical conversion capability was proposed [31]. Vapor–liquid–solid (VLS) epitaxial growth and focused electron beam-induced deposition were also researched as new processes to fabricate resonators [32].

This article aims to review the performance and the latest research progress of different resonant structures. MEMS/NEMS resonators have various structures, which can be used as mass sensing, oscillators, quantum spin coupling, filters, and gyroscopes. As the most common resonator in the MEMS field, the single or double-clamped beams resonant structure is introduced. Its excitation test methods, new manufacturing processes, research on resonance performance, and specific applications of resonators are described in detail. Furthermore, enumerate the hemispherical shell resonators, microdisk resonators, drum resonators, ring structure resonators, tuning fork resonators, SAW/BAW resonators, and introduce the design method of different structures, material characteristics, resonance performance, and application principles.

## 2. Manufacturing and Testing of Resonators

**Figure 1:**
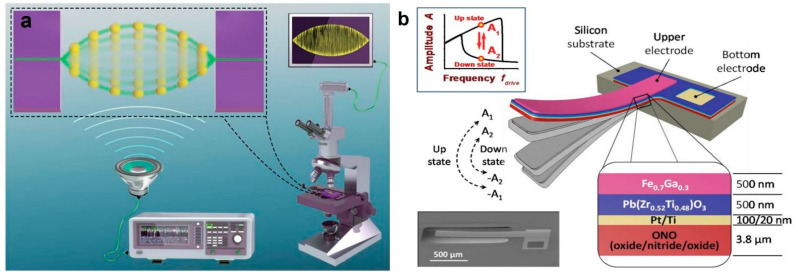
Acoustic and magnetoelectric coupling resonant excitation and test system. (**a**) Schematic of non-contact acoustic resonance test system. Adapted with permission from [35]. (**b**) Multi-layer heterostructure cantilever excited by magnetoelectric coupling. Top left image: SEM image of the hysteresis frequency response of multiferroic device; bottom left image: SEM image of cantilever beam. Adapted with permission from [39].

The excitation methods of single/double-clamped structures include acoustic drive, piezoelectric drive, laser drive [33], and magnetomotive transduction. Detection methods include radio signal detect, optomechanical detection [34], laser detection, and optical shadow detection. Small size high-frequency resonators such as carbon nanotubes have excellent electrical and mechanical properties and can work in the MHz to GHz range as resonators. However, due to impedance mismatch and parasitic capacitance, it is tough to measure the high-frequency electrical response of carbon nanotubes directly. Signal mixing technology is one of the few that use electrical detection to detect a frequency response of tens of GHz. Gouttenoire et al. achieved amplitude modulation (AM) and frequency modulation (FM) demodulation of suspended carbon nanotubes by tuning the mechanical resonance frequency, reducing noise and unwanted background signals [4]. In addition to using electrical test systems, it can also use optics or acoustics to achieve non-destructive testing. Bai et al. used a non-contact acoustic resonance test (ART) system to study the anti-fatigue properties of carbon nanotubes, as shown in Figure 1a. Compared with the electron microscope test system, the ARP system can achieve non-destructive detection of longer-sized samples. By depositing ${\mathrm{TiO}}_{2}$ on carbon nanotubes, changing the linear density to vary the resonance frequency [35]. Magnetomotive transduction technology can effectively support the testing of high-frequency devices [32]. Cleland et al. fabricated a few suspended silicon beams using photolithography and etching processes. Under the action of the magnetic field perpendicular to the beam, the beam is subjected to Lorentz force to generate electromotive force along the beam. The network analyzer measures the voltage at both ends of the beam [36].

Single crystal diamond (SCD) has excellent mechanical strength, thermal conductivity, electronic properties, and chemical inertness. However, due to the lack of shallow dopants necessary for high conductivity in diamond and the difficulty of heterogeneous growth of foreign substances on diamond, the realization of on-chip SCD NEMS/MEMS transducers has encountered a bottleneck. When synthesizing heterostructures from two-dimensional materials, the problem of lattice mismatch is also prone to occur [37]. Liao et al. proposed a self-sensing enhanced actuation (SEA) scheme to achieve energy conversion and realized a high-performance MEMS transducer. The fabricated SCD cantilever resonator places ultra-thin source-drain (SD) electrodes on the SCD resonator for self-sensing and places the gate (G) electrode vertically on the surface of the substrate next to the resonator for self-sensing drive. The heterodyne frequency downconversion method is used to characterize the resonant frequency of cantilevers. In the SEA scheme, the sensing part enhances the driving force, which in turn enhances its own sensitivity. The on-chip SCD NEMS/MEMS system shows the advantages of high self-sensitivity, low voltage drive, low power consumption, and high frequency (>100 MHz)( [38]).

The piezoelectric effect is also an important means to stimulate resonance. He et al. used a hybrid growth process to grow monocrystalline silicon nanowires on SOI as high-frequency NEMS resonators. The device was mounted on a piezoelectric ceramic with a resonant frequency of 18 MHz, and an AC signal was applied to directly couple to excite the resonance [40]. Onuta et al. fabricated a heterostructure cantilever beam using oxide/nitride/oxide (ONO) multilayer film buffer as shown in Figure 1b. Sputtering deposition of Pt/Ti double layer as the bottom electrode, deposit sol-gel $\mathrm{Pb}\left({\mathrm{Zr}}_{0.52}{\mathrm{Ti}}_{0.48}\right)O_{3}$ (PZT) multilayer on the bottom electrode, and magnetostrictive ${\mathrm{Fe}}_{0.7}{\mathrm{Ga}}_{0.3}$ is deposited under sputtering. There will be a strong magnetoelectric (ME) coupling in the range of $30–50{\;\mathrm{VOe}}^{-1}{\mathrm{cm}}^{-1}$ at the ${\mathrm{Fe}}_{0.7}{\mathrm{Ga}}_{0.3}$/PZT interface at the resonance frequency and excites the cantilever beam to resonate through AC magnetic field $H_{\mathrm{AC}}\;$or AC electric field${\;E}_{\mathrm{AC}}$ [39].

**Figure 2:**
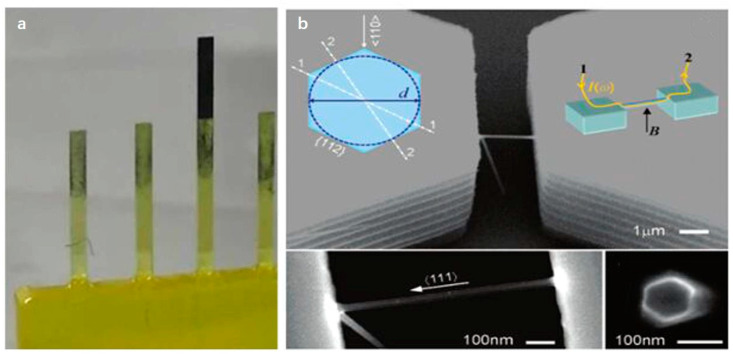
SEM image of single/double-clamped resonator. (**a**) Polymeric cantilevers (width 600 µm). Adapted with permission from [42]. (**b**) Silicon nanowires grown in micro trenches. Bottom left image: nanowires with a width of 600 nm are epitaxially grown along the <111> direction; bottom right image: hexagonal cross-section of silicon nanowire. Adapted with permission from [32].

Traditional manufacturing processes are subject to many limitations, such as complex processes, need expensive facilities and equipment, need to operate in an ultra-clean environment, and are not compatible with some flexible materials (polymers and plastics). In recent years, the additive manufacturing process, also known as 3D printing technology, has become a potential manufacturing method in the field of MEMS manufacturing [41,42]. It overcomes the manufacturing difficulties of many traditional processes. In particular, the traditional manufacturing process that wants to manufacture high aspect ratio structures can only be done by wet etching or deep reactive ion etching, while 3D printing can precisely control and manufacture complex structures with high aspect ratio. In 2013, Leary et al. first proposed the idea of 3D printing different parts or the entire microfluidic device to overcome the limitation of 2D MEMS channels [43], and the current microfluidic system is still the most suitable for 3D printing in MEMS. In the field of direct writing, other MEMS structures, such as microcantilevers, micro bridges, micro-coils, and freestanding structures can also be manufactured using 3D printing. 3D printing can use liquid, solid, or powder as feed materials. In 3D printing, the most promising technology is stereolithography (SL), which can use molten materials (liquid), radiation curable resins, or polymerizable materials as the main process feed. The light source is used to selectively cure these materials. If stereolithography is used in the micrometer range, this process is called micro-stereolithography (µSL). In a typical micro-stereolithography process, ultraviolet light is focused on a photopolymer layer with a thickness of up to 1–2 μm, so that sub-micron features can be engraved with high resolution [44]. Credi et al. successfully fabricated ferromagnetic microstructures based on polymer cantilevers through stereolithography, using layer-by-layer manufacturing methods and the self-supporting characteristics of resins, as shown in Figure 2a [42].

**Figure 3:**
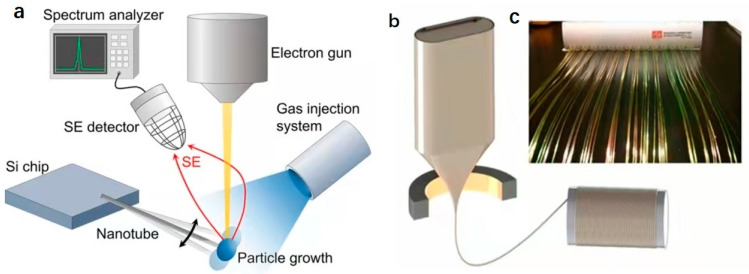
In-situ manufacturing and testing system of resonator and stretching process to manufacture MEMS resonator. (**a**) Schematic of particle deposition and in-situ measurement in a scanning electron microscope. Adapted with permission from [34]. (**b**) Schematic of the preform to fiber drawing process. Adapted with permission from [31]. (**c**) Flexible electrostrictive fiber array displays color reflection through the Bragg effect. Adapted with permission from [31].

In order to achieve a higher aspect ratio and more applications, other MEMS manufacturing processes are also constantly proposed. Arnold et al. used focused electron beam induced deposition (FEBID) to fabricate micro-cantilever resonators. This method can use metal/glassy carbon nanocomposites to manufacture resonators with arbitrary geometries with an aspect ratio greater than 100. Compared with the traditional manufacturing process, the large aspect ratio and excellent symmetry significantly reduce the transverse oscillations, and the sensitivity of the resonator can be improved due to Young’s modulus < 70 GPa [45]. Khudiyev et al. proposed a new method of manufacturing MEMS to improve aspect ratio, using preforms to produce fibers MEMS by a thermal drawing process as shown in Figure 3b,c [31]. The manufactured MEMS fiber device needs to realize the electromechanical conversion capability, so the material is selected as the electrostrictive material P(VDF-TrFE-CFE) compatible with the thermal drawing process, which has a stronger electric field and repeatable non-hysteresis response [31].

The traditional manufacturing process of MEMS devices uses photolithography [46] and etching to fabricate the resonant beam [47]. Compared with top-down photolithography and surface nanotechnology, bottom-up chemical synthesis technology has high crystal quality and a perfect termination surface. Feng et al. used vapor–liquid–solid (VLS) epitaxial growth to synthesize suspended silicon nanowires as shown in Figure 2b [32]. Traditional nanomanufacturing processes, such as reactive ion etching, are challenging to use for such small suspended structures without changing their sensing capabilities. Gruber et al. proposed a new nanofabrication method that can realize ultra-sensitive and functional carbon nanotube resonators in a scanning electron microscope. A carbon nanotube cantilever with a length of 1–15 μm is grown on a silicon substrate by chemical vapor deposition. A focused electron beam is then used to induce deposition to grow Pt particles on the top of the nanotube while detecting the secondary electron current. The secondary electron detector obtains the resonance spectrum of the noise-driven nanotube, thereby realizing the real-time mass of platinum particles as shown in Figure 3a [34].

## 3. Research on Resonance Performance

The primary characteristics of mechanical resonators are determined by the resonance frequency and quality factor (energy dissipation). At the intrinsic frequency of an ideal mechanical structure, the kinetic energy of a specific mechanical vibration is equal to the potential energy stored in the corresponding vibrational deformation of the structure. At the eigenfrequency, the total energy in the mechanical system is endlessly transferred back and forth between kinetic energy and potential energy. However, a real mechanical structure will have energy loss in each vibration cycle. In a real mechanical structure with inherent energy loss, the eigenmode mechanism is called resonance. The main frequency of energy exchange between kinetic energy and potential energy is called resonance frequency, which is usually close to the intrinsic frequency of the same system [48].

**Figure 4:**
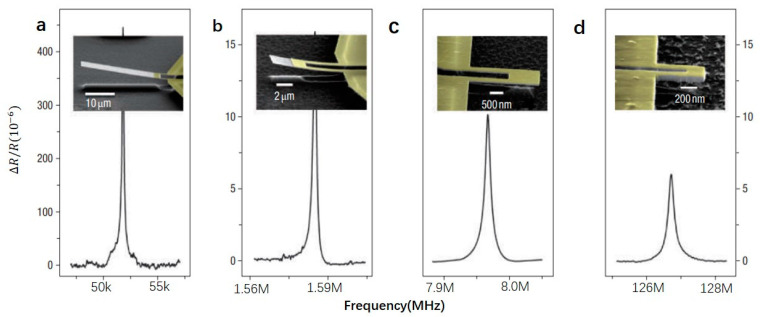
Fundamental-mode resonance frequencies of SiC nanobeams. Adapted with permission from [1]. (**a**) 52.1 k, 33 μm×5 μm. (**b**) 1.6 MHz, 10 μm×2 μm. (**c**) 8 MHz, 2.5 μm×0.8 μm. (**d**) 127 MHz, 0.6 μm×0.4 μm. The inset is the SEM image of cantilever beam.

Li et al. fabricated a few thin metal film nano cantilevers that work at very high frequencies as shown in Figure 4 [1]. The cantilever beam has a length of 600 nm–30 μm and a width of 400 nm–5 μm. Two narrow legs support it as an electrical path and strain concentrator. All cantilever beams are epitaxially made of 70 nm thick single crystal SiC and covered with a metal coating. By reducing the size of the resonator, the resonant frequency continues to increase. When the size of the resonator is reduced to 0.6×0.4×0.1 μm, the resonant frequency is up to 127 MHz [1]. In order to achieve a higher resonant frequency, Huang et al. fabricated doubly clamped resonators made of 3C-SiC film shows a high resonant frequency of 1.029 GHz, bringing NEMS resonators into the microwave field [49].

With the decrease of the thickness of the vibrating structure, the built-in strain of the structural material has more and more influence on the mechanical properties of the resonator. Most of the ultra-thin resonators work in the tension-dominated region, showing a strong dependence on aspect ratio *L*/*t*. In order to fabricate resonators with a modulus-dominant regime, Zhou et al. reported an ultra-thin, high aspect ratio, doubly clamped nanomechanical resonator with length L as high as 111 μm, thickness as low as 22 nm, and aspect ratio as high as *L*/*T*~ 5000. Under the axial strain of $6.3\times 10^{-8}$, the resonance frequency of the motion of multiple vibration modes at room temperature matches the predicted value of the Euler–Bernoulli beam theory very well. In order to approach the strain-free limit of ultra-thin nanomechanical resonators, independent ${\mathrm{Al}}_{2}O_{3\;}$tapes manufactured by atomic layer deposition (ALD) are transferred to pre-etched trenches. During the transfer process, the built-in tension caused by the manufacturing process is released, resulting in a resonant structure with ultra-low strain [50].

It is generally believed that high sensitivity requires high-quality factors, and peak position (and ultimate sensitivity) is affected by two factors: limited linewidth and signal noise. The former is determined by the quality factor of the resonator, and the latter is quantified by the signal-to-noise ratio. However, Roy et al. consider that the frequency fluctuation noise is proportional to the quality factor under low bandwidth without using high Q approximation and that the high damping system with full dynamic range should have better frequency stability (and sensitivity) than the same low damping system. In order to verify this hypothesis, a nano-optomechanical systems (NOMS) is used, in which a doubly clamped beam is coupled to a race-track optical cavity resonator and detected by a tunable diode laser. Through the temperature response of the resonator, the temperature resolution of 60 microkelvin at 300 Hz bandwidth is obtained, which provides a new idea for the application of high-performance ultra-sensitive resonators in a gas or liquid environment [51].

The nonlinearity in the resonator can be attributed to the coupling between the forced fields or the stretching of the midplane. The result of nonlinear effects is to make the resonator appear bistability, softening or hardening behavior, chaos, etc. In order to optimize the design and better understand the nonlinear dynamics, Tajaddidianfar et al. proposed the Homotopy Analysis Method (HAM) to derive the analytical solution of the frequency response for the resonator and proved that the second-order solution benefits from an adjustable parameter, which plays a key role in improving the accuracy of the analytical expression of the strongly nonlinear problem. Based on the analytical solution, the behavior of hardening, softening or mixed behaviors near the main resonance frequency is proposed [52]. Jin et al. proposed a nonlinear dynamic model based on the basic beam–photon–electron interaction and energy band theory to describe the cavity-free optical mechanical coupling. Through the theoretical model, the laser power and frequency can be tuned to cause the softening and hardening effect of the resonator to realize manipulation. Moreover, the dynamic response of the NEMS resonator, including various bifurcations, can be obtained by using parametric laser driving. It is found that the chaotic state can be controlled at several specific frequencies of the injected laser. Using a laser to control a nano resonator provides inspiration for exploring its nonlinear application [53]. In the carbon nanotube resonator, as the size is reduced to the molecular level, their vibration coupling is intensified, and there is a strong interaction, and even weak thermal fluctuations can cause the oscillator to appear nonlinear [54]. However, due to the small size of carbon nanotubes, there is no effective real-time detection method for the resonator. Barnard et al. used a high-precision micron-sized silicon nitride optical cavity as a sensitive photon microscope. Couple nano-scale carbon nanotubes to the optical cavity to realize the real-time measurement of the thermal vibration of the carbon nanotubes. By using a pair of electrically contacted gold micro tweezers to suspend and position a single carbon nanotube less than 100 nanometers away from the optical microcavity, enhanced optical coupling is achieved. With the high displacement sensitivity of $700{\;\mathrm{fm}\;\mathrm{Hz}}^{-1/2}$ and the high time resolution of the technology, it is found that the room temperature coherence is nearly three orders of magnitude longer than previously reported. It is found that the difference in coherence stems from the long-time non-equilibrium dynamics, which is similar to the Fermi–Pasta–Ulam–Tsingou recurrence in a nonlinear system. These experiments provide an integrated, sensitive, and high-bandwidth method for the detection of carbon nanotube resonators, and open up the study of nonlinear mechanical systems under the Brownian limit [55].

**Figure 5:**
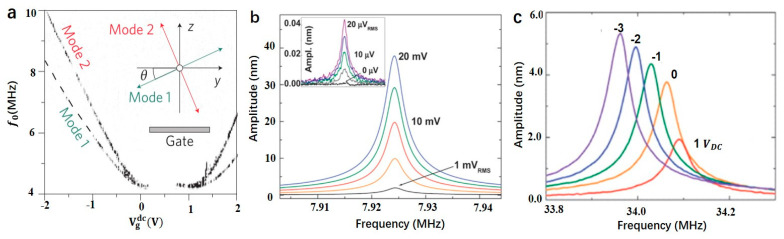
Voltage tuning of the mode, amplitude, and frequency of the resonator. (**a**) Mode splitting occurs when the gate voltage changes. Adapted with permission from [56]. (**b**) The amplitude of resonance depends on the change of AC voltage. Adapted with permission from [47]. (**c**) Fixed AC voltage, resonant frequency depends on the change of DC voltage. Adapted with permission from [47].

Dynamic modes of cantilever include transverse modes, torsional modes, lateral modes, longitudinal modes [2]. When driven by electricity, the electrothermal changes the strain of the resonator to realize the tuning of the natural mode and frequency of the resonator. Moser et al. designed a large displacement nanotube resonator with a spring constant as low as ~10 ${\mu N\;m}^{-1}$. Two modes appear in the resonator when the gate voltage is changed as shown in Figure 5a. The quality factor of mode 1 is *Q* = 48,000, and the quality factor of mode 2 is *Q* = 13,000 [56]. When the AC signal voltage increases, the nanowire’s effective stiffness decreases, and the resonance frequency declines with the increase in voltage [40]. Masmanidis et al. used the piezoelectric effect to fabricate the NEMS resonator. AC signal is used to drive the resonator, and DC voltage is used to adjust the depletion region width. The transverse electric field generates longitudinal strain in the cantilever beam, and the bending moment is generated due to the asymmetric distribution of the strain relative to the neutral axis of the beam leading to mechanical resonance. Since the strain is mainly concentrated in the depletion region of high resistance, the depletion region’s width can be adjusted by changing the AC voltage, thereby tuning the piezoelectric efficiency to change the amplitude of the resonator. At the same time, a double clamped cantilever beam, which can be used with DC bias tuning the resonance frequency as shown in Figure 5b,c [47]. In order to solve the problem of thermal expansion coefficient mismatch, Guzman et al. proposed to use SiC as an independent resonance and heating element to achieve frequency tuning [57]. For cantilever beams with strong magnetic and electrical coupling, the piezoelectric voltage caused by the strain enhances multiferroic signal transduction. $H_{\mathrm{DC}}\;$is used to tune the resonant frequency. Due to ME coupling, the piezoelectric signal also changes with the change of the resonant frequency [39]. Jia et al. report the most flexural-mode micromechanical resonators working in liquids. The microdisk resonator is made of silicon carbide and is characterized by its multimode resonance in air and water. Up to 28 flexural modes were observed in the air and 12 modes in water, with frequency ranges of ~3–190 MHz and $Q_{s}\;$up to ~30 [58].

The quality factor defines the rate at which the nanomechanical resonator consumes energy. The energy dissipation methods include the interaction loss with the surrounding liquid or gas medium, the clamping loss of the elastic wave propagating to the substrate, or the inherent consumption of the resonator such as thermoelastic loss. Dispersion mechanism. Most of the micromechanical resonators need to achieve high quality factors to be applied [48].

In order to improve the quality factor of the resonator and reduce the energy loss, Aykol et al. fabricated suspended carbon nanotubes grown on top of metal electrodes and studied the resonance characteristics of being clamped by weak van der Waals forces. Since the clamping conditions remain unchanged, even if the temperature rises, the nanotubes are separated from the sidewalls and still do not affect the quality factor [59]. Another massive loss of the resonator comes from the working fluid environment. When the resonator resonates in liquid, more significant energy loss occurs due to viscous damping. Verbridge et al. manufactured single clamped and double clamped SiN flexural nanoscale resonators and placed them in air, alcohol, water, and buffer for resonance [33]. For a double clamped beam with a length of 2 μm, a width of 165 nm, and a thickness of 125 nm, the resonance frequency in air is 145 MHz, and the quality factor is about 400. The resonance frequency in water drops to 95 MHz, and the quality factor drops to about 5 [33].

Elastic strain engineering (ESE) can use stress to tune material properties. In mechanical engineering, the complementary strain engineering technique “Dissipative dilution” is proposed to reduce the dissipation of resonators, and the dissipative dilution can be improved with the decrease of device size, which means that resonators with smaller mass can have a higher quality factor. Thus, the stiffness of the stressed material is effectively increased without increasing the loss. The uniform beam model shows that the *Q* of the fundamental mode (*n* = 1) is usually the highest, and increasing the aspect ratio or stress can increase the Q value. Ghadimi uses the “soft clamping” mode and non-uniform phononic crystal (PnC) mode to obtain the ideal performance of clamp-free resonator and calibrates the strain and bending motion of self-supporting ${\mathrm{Si}}_{3}N_{4}$ nanobeam. Therefore, more than 800 million quality factors were obtained at room temperature [60].

Bao et al. proposed a strategy to reduce energy dissipation by using a suspended frame structure and phononic crystals (PnC) to realize a silicon-based MEMS resonator with a high quality factor. The resonator body of the resonator is composed of AlN-ON-SOI, the suspension frame structure isolates the mechanical vibration between the resonator body and the anchoring substrate, and the PnC array is used as a frequency selective reflector to reduce energy leakage. Experimental results show that the quality factor of the proposed resonator is improved by 7.8 times and 1.5 times, respectively, compared with the bare resonator and the resonator with only a suspended frame structure [61]. Miao et al. designed a method to tune the effective quality factor ($Q_{eff}$) through mechanical pumping. The red (anti-Stokes) and blue (Stokes) sidebands are activated to transfer coherent energy in the coupled mode. The mechanical pump does not require mechanical design and processing and can be flexibly realized by adding a $V_{p}\cos\left(\omega_{p}t\right)$ electrical signal. The $Q_{eff}$ of the coupled MEMS resonator can be adjusted considerably. The results show that the decay time of the resonator can be greatly increased to 60.54 s, which is equivalent to a million-order (1.32 million) $Q_{eff}$ [62].

## 4. Application of Resonant Sensor

### 4.1. Single or Double-Clamped Resonators

#### 4.1.1. Resonators in Micromechanical Thermal Analysis

Thermal analysis refers to the use of temperature control and other techniques to study the thermal properties of the sample material, such as the glass transition temperature or melting temperature of polymers [63] or proteins [64]. Due to its high sensitivity and short thermal response time, the micromechanical resonator can be used for micromechanical thermal analysis (MTA) of nanogram samples. Nguyen et al. fabricated a conductive pyrolytic carbon resonator for thermal analysis of polymer nano samples. The change in resonance frequency obtained by spraying deposited polymer can be used as a mass sensor. At the same time, the resonator is used as a resistance heating element, and the glass transition temperature of semicrystalline poly(lactic acid) (PLLA) and the melting temperature of poly(caprolactone) (PCL) are obtained by the change of resonant frequency and quality factor after heating [65]. Thermomechanical analysis of the phase transition characteristics of PLLA, when the temperature of the resonator increases, the resonance frequency of PLLA decreases due to the positive thermal expansion coefficient, while the glass characteristics emerged after 60 °C, Young’s modulus increases, and the resonance frequency is determined by the negative thermal expansion coefficient of pyrolytic carbon starts to rise. The change of the quality factor is also related to the viscous damping in the polymer phase transition [65].

**Figure 6:**
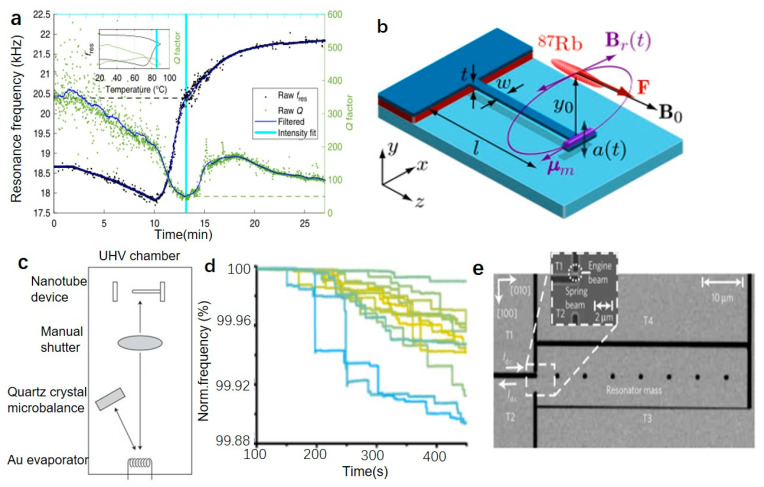
Single/double-clamped resonators as sensors. (**a**) Resonant frequency and quality factor of TP MH particle cantilever during thermal cycling. Adapted with permission from [67]. (**b**) Schematic diagram of BEC resonator. Magnetic coupling between atom and nanoresonator. Adapted with permission from [21]. (**c**) Nano-resonant mass spectrometry test system, Au atoms evaporate in the UVH cavity, manual shutter controls mass deposition and closing, QCM measures mass flow. Adapted with permission from [81]. (**d**) Frequency-time trace diagram obtained by 15 nanometer resonant arrays after 10 min of acquisition. Adapted with permission from [86]. (**e**) SEM image of the heat engine. The inset is an enlarged view of the engineering beam and spring beam. Adapted with permission from [91].

In thermomechanical analysis, there is a trend to use NEMS [66] and MEMS to analyze smaller samples, which promotes the use of these methods in drug research. Due to the great challenge of placing different samples on the resonator, a single sample particle can directly be made into a resonator with a specific shape to realize resonant. Okeyo et al. proposed a “Particle Mechanical Thermal Analysis” (PMTA) method, which uses a single particle as a resonator to explore changes in mechanical properties during thermal cycling. The two model particles are elongated particles of theophylline monohydrate (TP MH) and a rolled sheet of collagen particles. Since TP MH undergoes a phase change during dehydration and can be monitored with a thermal microscope, it can be used as a simple verification for PMTA analysis of particles. For TP MH particles with a size of 1000×65 μm, the resonance frequency is 19±3.8 kHz. In the thermal cycle test of 25–90–25 °C (5 °C/min), the resonance frequency changes significantly after 11 min as shown in Figure 6a. The first derivative shows that the sudden change of the resonance frequency is due to the main phase transition at about 79 °C, while the fluctuation of the resonance frequency is mainly due to the change of Young’s modulus caused by particle softening or water loss [67]. The change of quality factor is also related to the softening and water loss of particles, but the total is more significant than 100 [67].

#### 4.1.2. Resonators in the Quantum Field

Atomic chips are very suitable for the realization of hybrid quantum systems [68]. Since the resonator’s position measurement as a sensing element is ultimately limited by quantum mechanics, it is a long-term goal to detect the influence of quantum mechanics on the micromechanical oscillator [69]. Knobel et al. designed a double-clamped GaAs beam resonator to detect the influence of quantum mechanics on macroscopic mechanical oscillators. When the temperature of resonators is lower than the energy quantum $T\ll T_{Q}$, the oscillator energy begins to quantize and produce an internal fluctuation amplitude. Using single-electron transistors to test the resonance characteristics can achieve a sensitivity of the quantum-limited. The experimental device is installed on a dilution refrigerator, and the temperature is less than 30 mK. An Al electrode is used to form a single-electron transistor (SET) and beam electrode, and the capacitance coupling between SET and beam is used to measure the movement of resonators. The resonance frequency is 116.7 MHz, the quality factor is 1700, and the measured displacement sensitivity is $2\times 10^{-15}\;{\mathrm{mHz}}^{-1/2}$ with a factor of 100 larger than the quantum limit [70].

The phonon band gap structure can be used to change the emission or scattering of phonons, which can reduce the thermal coupling with the surrounding environment by acoustic radiation in quantum optomechanical and electromechanical experiments [71]. Maccabe et al. proposed to measure the phonon lifetime at microwave frequency by combining the phonon bandgap acoustic shield and nano-scale silicon acoustic cavity. The nanocavity is formed by an optical mechanical crystal (OMC) nanobeam resonator, which supports the acoustic breathing mode of $\omega_{m}/2\pi$ ≈ 5 GHz and the colocalized optical resonance mode of $\omega_{c}/2\pi \approx 175\;\mathrm{THz}$. In order to minimize the mechanical clamping loss, the nanobeam is anchored to the silicon body with a periodic cross structure [72]. Using the colocalized optical mode of the cavity excited by a pulsed laser, the single phonon sensitivity is measured as low as millikelvin temperature, and the phonon lifetime is as high as$\;\tau_{ph.\;0}\approx 1.5\;s$. These acoustically engineered nanostructures provide a window to understand the material sources of quantum noise and have potential applications in quantum mechanical testing and quantum memory devices [73].

Quantum control of spin and mechanical degree of freedom can be realized by coupling electron spin to the resonator with a high quality factor. As cantilever and doubly clamped beams are relatively easy to manufacture and characterize, it is very convenient for spin-mechanics research [74]. Carter et al. demonstrated the strain-induced coupling between the hole spin in quantum dots and the mechanical motion of the cantilever. By measuring the spin splitting of electrons and holes, it is shown that the change of the hole spin is much higher than that of the electron spin due to the stronger spin orbit interaction [75]. Oeckinghaus et al. connected the spin with the coupled cantilever system and showed their correlation. In addition, the coherent spin–spin coupling induced by common mode is analyzed, and the entanglement generation between long-distance spins is estimated [76].

Quantum state nanomechanical oscillators can be coupled with phase qubits [77]. If the resonator wave function can be manipulated coherently, extending the pioneering experiment of trapped ions [78] to macroscopic objects is possible. The perfect photostability of nitrogen vacancy (NV) defects at room temperature can be used as a useful single-photon source, manipulated by resonant microwave excitation, and optically read out its ground state spin-triplet state. Arcizet et al. used the NV center located in the diamond crystal as a single-photon source to study the nanomechanical oscillator dynamics. The nanomechanical oscillator is composed of silicon carbide nanowires attached to the end of conductive tungsten. Time-resolved nanocrystal fluorescence and photon correlation measurements are used to detect the dynamics of the nanoresonator. When the system is placed in a strong magnetic field gradient, magnetic coupling is induced between the nanomechanical oscillator and the NV electron spin, and resonant optical. It is proved that the resonant optomechanical coupling will not reduce the intensity when the sub-wavelength size resonator interacts with the optics [79]. The micro-and nano-structured condensed matter system enters the state described by quantum optics, and it will be possible to realize precise measurement and quantum information processing [80]. Treutlein et al. used cantilever beam resonators to study the magnetic coupling between atomic spins in Bose–Einstein condensate (BEC) and the single vibration mode of nanomechanical resonators. The tip of the cantilever is equipped with a single domain Co magnet. The magnet generates a magnetic field above the tip of the resonator, and ^87^Rb atoms are trapped in a magnetic microtrap above the resonator as shown in Figure 6b [21]. Since the magnetic field can tune the resonant frequency $w_{L}$ of the atom, the magnetic decoupling $\delta =w_{r}-w_{L}$ between the resonator resonant frequency $w_{r}$ and the atomic resonance can be regulated. When δ is equal to 0, the coupling makes the atom spin flip, which can detect the thermal oscillation of the cantilever with the atoms. The BEC on the atom chip can be used for sensitive probes, coolants, and other applications [21].

#### 4.1.3. Resonator as Mass Sensor

Nanomechanical resonators can be used as precision mass sensors, and the particles are adsorbed on the resonator beam because of the different mass and position, which causes the resonant frequency of the resonator to shift [81]. The relationship between the shift of the resonance frequency Δf and the particle mass and the intrinsic mass of the beam$\;m_{0}$ is

(1) $$\Delta f=-\frac{f_{0}}{2m_{0}}\Delta m$$

where $f_{0}$ is the resonant frequency of the beam and the change in mass Δm is a function of particle adsorption position [81]. Yang et al. designed a double clamped beam made of SiC epitaxial layer. The manufactured nano double-clamped beam resonator can be used as a mass sensor to achieve high resolution. The mass sensing device performs in-situ measurement in an ultra-high vacuum of $<10^{-10}\;\mathrm{Torr}$ and a highly controllable flux of Xe atoms or $N_{2}$ molecules are delivered to the surface of the equipment through a mechanical gas nozzle. The device can realize the in-situ real-time measurement of mass, and the best mass resolution obtained is ~7 zg, which is equivalent to ~30 Xe atoms or a molecular mass of 4 KDa [82].

In order to reduce the mass of the resonator to improve the sensitivity of the resonator effectively, Jensen et al. used a single-clamped carbon nanotube with a high elastic modulus as resonators, and its mass was only about $10^{-21}\;\mathrm{kg}$ as shown in Figure 6c [81]. Single-clamped carbon nanotubes have a higher dynamic range than a double-clamped geometry and have a higher quality factor due to low clamping loss. Furthermore, use an advanced radio receiver to detect its resonance, broadcast a radio signal to the nanotube and use its field emission characteristics for testing. The fundamental frequency of the resonator is around 328.5 MHz, and the mass sensitivity is $1.3\times 10^{-25}{\;\mathrm{kg}\;\mathrm{Hz}}^{-1/2}$, which is equivalent to 0.4 gold atoms ${\mathrm{Hz}}^{-1/2}$ [81].

Compared with piezoelectric resonators measuring masses [83] greater than picograms, mass spectrometry (MS) is 12 orders of magnitude lower on the mass scale, but due to the impact of ionization yield and ion transfer-related losses, mass spectrometers will consume longer time and more samples [84]. Dominguez-Medina et al. proposed a nanomechanical resonance test system that can analyze the mass in the range of MDa~GDa. The detector consists of 20 nanomechanical resonators arranged in a 4×5 array. Each nanoresonator includes a fixed-free lever beam, and two piezoresistive pressure gauges with p++ doped silicon, connected to the cantilever beam to measure the movement of the cantilever [85]. Driven by electrostatic force, the average value of the resonator resonance is 19.16 MHz, the maximum deviation between each resonator is only 2%. The measurement system uses surface acoustic wave nebulization of the analyte from the solution under atmospheric pressure, uses the inertia of the particles to transfer and focus effectively, and determines the mass of a single particle by using an array of nanomechanical resonators in a high vacuum. The frequency-time traces obtained by 15 nano-resonant arrays after 10 min. The step jump in Figure 6d [83,86] corresponds to the landing of nanoparticles. The system can measure the mass of empty or DNA-filled bacteriophage T5 capsids with masses up to 105 MDa at a resolution higher than 100 [83,86]. Sage et al. reported a NEMS-MS device that uses individually addressed nanomechanical resonator arrays. Thanks to the increase in the capture area, the mass spectrometry analysis time is decreased with more than one order of magnitude. Each resonator is designed with a different resonant frequency that can be individually addressed, and the input and output ports of each resonator are interconnected. Twenty resonators are arranged in a 5×4 array. Each resonator is a typical double clamped beam. Using this individually addressed resonator array can obtain mass spectrometry analysis of metal aggregates in the MDa range [87].

Reducing the size of the nanomechanical resonator has superior performance as a mass sensor. However, reducing the size will reduce the surface area for analyte adsorption, thereby reduce the adsorption capacity. Venkatasubramanian et al. proposed a locally porous nano cantilever made by lithographically controlled stain etching. It can measure volatile organic compounds. Due to the increase in mass responsiveness and adsorption mass, the porous structure increases the sensing signal by an order of magnitude, and the limit of detection (LOD) is also increased by six times. Use electron beam lithography to define the device structure and $V_{2}O_{5}$ as an oxidant to control the porosity and pore depth. When the maximum oxidant concentration is set to 228 mM, a device with maximum porosity and a depth of about 80 nm can be obtained within a specific time. At atmospheric pressure and a temperature of 298 K, the non-porous nanomachine cantilever beam’s resonance frequency is 10.45 MHz. Under the same conditions, the frequency decreases as the porosity of the nanomachine device increases [88].

In addition to frequency shift response, mass sensing can also be achieved by mode localization. Mode localization is a phenomenon first discovered by Anderson in periodic systems [89]. It describes the rule that the vibration energy can gather in some regions of the system when there are small disturbances. Theoretical analysis and experimental verification show that the eigenstate shift (amplitude shift) is 3–4 times larger than the corresponding resonance frequency under picogram-order mass disturbance. This kind of sensor can provide an important advantage for inherent common mode suppression, making it less susceptible to false positive readings caused by frequency shift based sensors. In addition, two cantilevers with different geometry can be coupled to form a synchronous oscillator. By applying a small mass disturbance on the tip of the low-frequency cantilever, the picogram magnitude mass sensing can be realized [90].

#### 4.1.4. Self Oscillating Resonator

The silicon engine beam can drive the silicon resonator to produce sustained oscillation due to the feedback mechanism of silicon’s inherent piezoresistive heating and thermal expansion. As an engine, its power density is almost 1000 times that of modern automobile engines. Steeneken et al. designed a silicon crystal driven by DC as a cyclic heat engine, using a silicon engine beam to drive resonator to produce sustained oscillation. The resonator structure consists of a mass of $12.5\times 60.0\times 1.5{\;\mu m}^{3}$, including a 3 μm wide and 800 nm long spring beam and a 280 nm wide and 800 nm long engine beam as shown in Figure 6e. The resonator uses the AC electrostatic terminal T3 to drive the heat engine and uses the piezoresistive effect to detect the strain change in the heat engine beam, and the resonance frequency is 1.26 MHz. As a piezoresistive heat engine, disconnect the voltage terminal T3 and connect the device to a DC source and a capacitive coupling oscilloscope only at the terminal T1. When the DC increases above the threshold $I_{t}=1.19\;\mathrm{mA}$, significant spontaneous oscillation will occur, and a sinusoidal output voltage with a frequency of 1.26 MHz will be generated [91]. ${\mathrm{VO}}_{2}$ material undergoes a significant electrical nonlinear change during phase transitions, which leads to a periodic instability state of current/voltage under constant electrical bias. Manca et al. designed ${\mathrm{VO}}_{2}$ coupled electronic and structural phase transitions to achieve local self-excited resonant motion as oscillators. ${\mathrm{VO}}_{2}$ will undergo phase transitions above 65 °C, and the conductivity will be significantly enhanced. In order to avoid the massive frequency shift of the mechanical mode caused by the phase transitions of the full-bridge, a small ${\mathrm{VO}}_{2}$ active area is reserved in the gap of the bridge to cause the electrode to generate Joule heat confined in this area. A much smaller resistance $R_{0}$ forms a specific combination with the microbridge’s resistance. As the microbridge’s temperature increases, the resistance decreases so that the voltage on $R_{0}$ increases, leads the Joule heating of the microbridge decreases, and then the resistance increases, and a new cycle begins to generate relaxation oscillations [92]. The voltage $V_{0}$ is used to control the relaxation oscillation frequency to achieve a gain of 250 kHz/V. For an oscillation of f=32.6 kHz, the jitter amplitude is about 4–5% [92].

### 4.2. Hemispherical Shell Resonators

**Figure 7:**
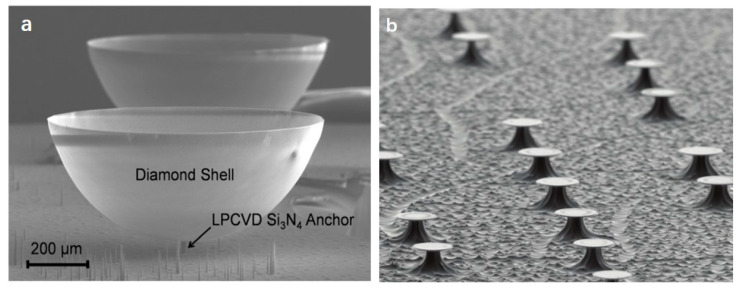
Hemispherical shell resonator and microdisk resonator made of diamond material. (**a**) SEM image of diamond shell resonator with ${\mathrm{Si}}_{3}N_{4}$ as anchor. Adapted with permission from [93]. (**b**) SEM images of inkjet-printed diamond micro-resonators. Adapted with permission from [24].

The hemispherical shell resonators have excellent performance and structural symmetry and can be used as a high-precision and high-reliability gyroscope. Thin-film polycrystalline diamond has a low surface loss and ultra-low thermal elastic damping due to its excellent strength to the density ratio and surface properties, making it ideal for manufacturing resonators. Heidari et al. used micro-electro discharge machining to manufacture a hemispherical silicon mold and fabricate a 1.1 mm diameter diamond wineglass resonator made of ${\mathrm{Si}}_{3}N_{4}$ as anchors as shown in Figure 7a [93]. Piezoelectric and electrostatic excitation are used, respectively, and optical detection is applied. The two 2*Θ* elliptical vibration modes with frequency $f_{1}=18.316\;\mathrm{kHz}$ and $f_{2}=18.321\;\mathrm{kHz}$ are obtained, the minimum frequency mismatch is 5 Hz, and the quality factor measured is 20,000. By studying the influence of the resonator’s geometry on the resonant frequency, it is found that the absolute frequency of the resonator increases linearly with the increase of the anchor diameter [93]. Due to the higher surface tension of metal, it is superior to achieving a highly symmetrical structure. Using bulk metallic glasses (BMG), such as ${\mathrm{Pt}}_{57.5}{\mathrm{Cu}}_{14.7}{\mathrm{Ni}}_{5.3}P_{22.5\;}\;$materials, and TPF (thermoplastic forming) process, Kanik et al. fabricated a wineglass hemispherical shell resonator. The resonator’s diameter is 3 mm, the surface is super smooth, and the structure is highly symmetrical. The resonance frequency of 13.944 kHz is obtained in a degenerate mode of *n* = 2 by optical detection, and the quality factor is about 6200 [13].

### 4.3. Microdisk Resonators

A microdisk resonator has a large capture area, considerable rigidity, and proper symmetry, and therefore has a high operating speed [14]. Furthermore, a microdisk resonator has a resonance frequency exceeding gigahertz and moderate fluid dissipation and can also be used in liquid research. Microdisk resonators can be made of different materials that have different characteristics and applications. GaAs is a low-dissipation, high-purity crystalline material, and its epitaxial control can be used to manufacture optomechanical heterostructures [94,95]. SiC material has excellent thermal conductivity, excessively high elastic modulus, and chemical inertness [14]. Nanocrystalline diamonds are ideal materials for manufacturing various microelectromechanical devices and mass sensors due to their high-frequency working range and excellent material properties [24].

Optomechanical disk resonator has many characteristics, such as ultra-high resonant frequency, large sensing area, ultra-high displacement sensitivity, and low mechanical energy dissipation. The low-frequency vibration modes of proteins, viruses, bacteria, and other nano biological particles involve coherent collective vibration in terahertz and gigahertz domains. Gil Santos et al. measured the characteristic frequency and mechanical loss (quality factor) of low-frequency vibration mode of bacteria using an optomechanical disk resonator supporting mechanical radial breathing mode and optical whispering gallery modes. The radial displacement of the disk changes the electromagnetic condition of the disk to change whispering gallery mode optical resonances and cause the change of the output light intensity. The small Brownian fluctuations associated with the rigid radial basis function can be solved by strong optomechanical coupling, which provides a displacement noise base of ~$10^{-18}{\mathrm{mHz}}^{-2}$. When S. epidermidis bacterium was deposited on an optical mechanical disk by an electrospray ionization system, the resonance frequency of a disc with a radius of 2.5 μm (frequency 546 MHz) was divided into two broad and near resonants. When the frequencies of the disk and bacteria are similar, their vibration modes will be hybridized. Resonance broadening also indicates that the presence of bacteria increases a lot of mechanical loss. By developing a general theoretical framework to describe the coupling, it shows that UHF optical mechanical resonators can be used for vibration spectrum analysis, and have the unique ability to obtain the information of a single biological entity [96].

Gil-Santos et al. used aluminum gallium arsenide (AlGaAs) as a pedestal to fabricate a GaAs microdisk resonator with a thickness of 320 nm and a disk radius of 1–3 μm. Monochromatic light with a wavelength of 1.3 μm is coupled to the vicinity of the resonator through a GaAs waveguide to achieve frequency measurement. Due to the large refractive index of GaAs, the disc/waveguide structure can be immersed in a transparent liquid and maintain its light guiding properties [97]. Wang et al. proposed a microdisk resonator detection platform using a resonator made of silicon carbide material. An ultra-sensitive scanning laser detection scheme is used to achieve a high-sensitivity readout of multi-mode resonance, and the displacement detection sensitivity can reach ~7–14 ${\mathrm{fm}\;\mathrm{Hz}}^{-1/2}$. By focusing the laser spot on a given position of the device, all modes within a wide range of frequencies can be obtained, and then a single resonance mode can be focused on by scanning the full range of the device. The device’s defects and asymmetry can be characterized by the accurate detection of a particular resonance mode. For resonators with up to nine resonance modes, the higher vibration mode tends to have a smaller thermomechanical amplitude. The asymmetrical mode shape has a lower quality factor than its conjugated mode shape [14]. Sartori et al. proposed a low-cost inkjet-printed method for manufacturing diamond micro-resonators, which can realize large-area manufacturing without masks. The water-based diamond ink is printed on the silicon wafer in the form of 50–60 μm spots, and a uniformly shaped disk with a diameter of about 38 μm is printed, and then the nanocrystalline diamonds ink is grown into a 1 μm thick diamond film disk by chemical vapor deposition. The microdisk is suspended by reactive ion etching to obtain a disk resonator as shown in Figure 7b. The resonant frequency obtains in the range of 9–30 MHz by laser interferometry, the quality factor exceeds $10^{4}$, and the $\left(f_{0}\times Q\right)$ figure in a vacuum is as high as ≈$2.5\times 10^{11}$ Hz. After depositing gold, it can be used as a mass sensor to achieve $981{\;\mathrm{Hz}\;\mathrm{fg}}^{-1}$ sensitivity [24].

### 4.4. Drum Resonators

**Figure 8:**
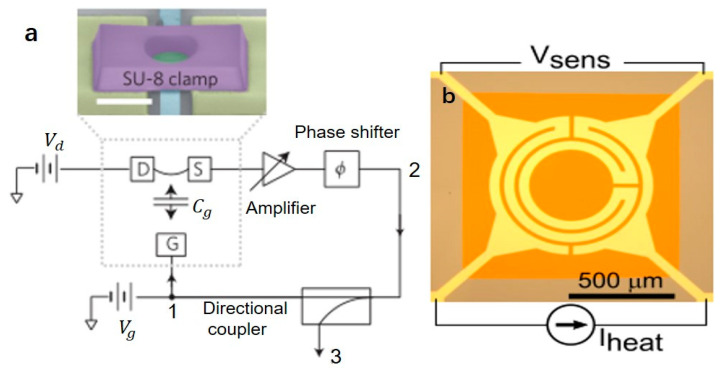
Drum resonator. (**a**) Simplified circuit diagram of graphene oscillator. Inset: false-color SEM image of graphene drum resonator with SU-8 clamp. Adapted with permission from [99]. (**b**) SiN membrane resonator with integrated Pt heaters and schematic of the electrical heating circuit. Adapted with permission from [23].

The drum resonator is usually a thin-film structure, and the surrounding is clamped, which can realize sensing and electrical applications, especially because of its high-frequency characteristics, it can be used as an octave frequency tuning, high-frequency random switching, and self-oscillation in the circuit. Graphene has low mass and low stiffness with superior mechanical properties [98]. As a membrane resonator, it is an atomic-level ultra-thin and super-strong material that can resonate at megahertz to achieve high stochastic switching rates [26]. At the same time, its high-frequency resonance can be tuned by applying voltage. Chen et al. designed a circular graphene drum oscillator with a local gate electrode suspended on an insulating substrate. The oscillator is composed of a graphene suspended strip, metal electrodes, and a clamping structure made of SU-8 epoxy photoresist as shown in Figure 8a [99]. The clamping structure increases the stiffness of graphene and eliminates the complex vibration mode at the edge of graphene, and limited the size of the oscillator to 2–4 μm. The graphene is driven to vibrate by applying a DC and RF bias to the gate, and a second DC bias is applied to the drain to read. The resonant frequency of graphene can be tuned up to 14% through electrostatics. In turn, the resonant motion will also adjust the charge density, thereby adjusting the conductance and leakage current. A variable gain amplifier is used to set the resonance gain, and an adjustable phase shifter is used to adjust the phase to zero, to meet the Barkhausen criterion to realize self-oscillation [99]. Dolleman et al. designed a single-layer graphene drum resonator as a high-frequency stochastic switch. Since the switching rate follows Kramer’s law and increases as the effective temperature increases, using a VNA to drive the membrane at a fixed frequency and using an arbitrary waveform generator (AWG) to add random fluctuations to the resonator artificially will increase the effective temperature, and increasing the fluctuating power will increase the switching rate. A switching rate of up to 4.1 kHz is obtained at room temperature, which is more than 100 times higher than that of existing mechanical resonators [26]. In order to detect the in-plane stress of multilayer graphene in real-time by measuring the resonant frequency of the resonator, Robinson et al. fabricated a nanomechanical drum resonator with a diameter of 1–4 μm. For the purpose of exploring the influence of the evolution of defects on the mechanical properties of graphene, the resonator was exposed to 300 eV of argon ions to introduce defects [100]. After the resonator with a diameter of 4 μm is implanted with argon ion defects, the resonant frequency will increase from 60–75 MHz to 100–125 MHz [100].

The powerful controllable interaction between quantum systems can bring many breakthroughs in the field of quantum science. Karg et al. proposed to connect two systems [101] with laser beams in annular geometry. Through the loop, the system can exchange photons and achieve two-way interaction to achieve long-distance Hamiltonian interaction. For any two systems coupled to the light in opposite phases, the quantum noise cancels the interference and the decoherence is suppressed. In this way, the coupling system can be effectively isolated from the environment, even if the light field plays a strong interaction between them. Using this scheme to couple a collective atomic spin and a micromachined film, which are kept in different vacuum chambers, a hybrid atomic optomechanical system is realized. The mechanical oscillator is the (2,2) square drum mode of silicon nitride film, and the vibration frequency is 1.957 MHz, the quality factor is $1.3\times 10^{6}$. It is placed in a short single-sided optical cavity to enhance the optical mechanical interaction while maintaining a large cavity bandwidth for fast and effective coupling to the external light field. One of the characteristics of spin system is that it can realize such an oscillator with positive and negative effective mass. This property enables to realize different Hamiltonian dynamics by using the spin coupled with the membrane. The coupling is highly adjustable. It promotes the reliable processing of quantum information and generates entanglement between spatially separated quantum processors at room temperature [102].

St-Gelais et al. designed SiN drum resonators for an octave frequency tuning device as shown in Figure 8b [23]. The resonator is composed of a 1.05 mm wide and 100 nm thick low-stress SiN film. A heating current is applied to a 50 nm thick labyrinth platinum resistor deposited on the film to reduce the film’s tensile stress to lower the resonance frequency and achieve frequency tuning. As the heating power increases for different mechanical modes, the resonance frequency decreases. The adjustment of the heating power to the resonant frequency exceeds one octave. For example, as the heating power increases for the fundamental mode, the resonant frequency can be reduced from 121.1 kHz to 56.1 kHz. Due to the material damping in the metal film, the quality factor decreases when the tensile stress is reduced [23].

### 4.5. Ring Resonators

**Figure 9:**
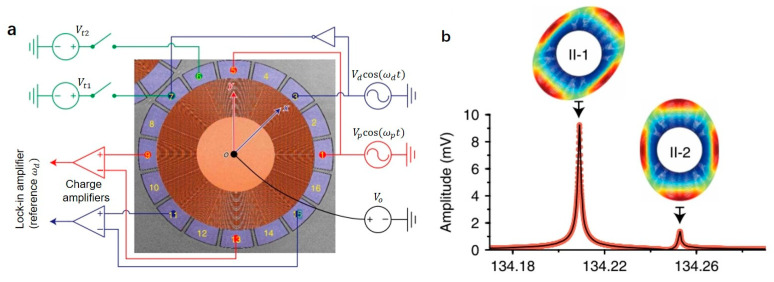
(**a**) The experimental platform of ring resonator gyroscope. Uniformly distributed capacitor electrodes to achieve driving, tuning, and detection. (**b**) Measured mechanical mode of the resonator. Adapted with permission from [27].

Ring resonator has a vital application in gyroscope. Different modes of ring resonance can be produced by using driving and tuning electrodes. When the driving mode matches the detection mode, high-performance measurement and stability can be achieved. Zhou et al. designed a capacitive symmetrical microelectromechanical ring resonator test bench. The resonator is made of highly doped P-type (111) single crystal silicon, with a diameter and thickness of 720 and 40 μm, respectively, and is supported by a central anchor. It contains 45 nested rings with equal intervals of 3 μm. Spokes interconnect the adjacent rings. Sixteen capacitive electrodes surround the resonator for excitation, sensing, and tuning in-plane response. The capacitive electrode introduces a nonlinear electrostatic field around the mechanical resonator, resulting in an inhomogeneous force term, a stiffness correction term, and the interaction between multiple nonlinear modes. Two pairs of degenerate modes were tested at room temperature, $f_{\mathrm{II}-1}=134,209\;\mathrm{Hz}$, $f_{\mathrm{II}-2}=134,253\;\mathrm{Hz}$, $f_{\mathrm{III}-1}=166,498\;\mathrm{Hz}$, and $f_{\mathrm{III}-2}=166,949\;\mathrm{Hz}$. The parametric pump-induced dynamic operation can study the coupling between linear and nonlinear modes. The specific operation is shown in Figure 9 [27]. In addition to applying drive signals to electrodes 3 and 7, pump signals are applied to electrodes 1 and 5. The realization of sideband coupling can dynamically adjust the coupling stiffness between modes, and the energy transfer between modes can even increase or decrease the quality factor of a specific mode [27]. For the ring resonant gyroscope, reducing the ring width and increasing the outer radius can increase the quality factor of the resonator. Li et al. proposed to optimize the design of the spoke length distribution of the traditional resonator or to optimize the mass distribution by suspending the lumped mass on the ring frame, and achieve a resonator with a very high-quality factor by decoupling stiffness. The quality factor measured by the ring-down technique is as high as 510,000, achieving a new record of 74.9 s longest decaying time constant. The use of stiffness decoupling design increases the quality factor and decaying time constant by 112% and 432%, respectively. The resonant frequency of electrostatic driving and detection is about 2163.8 Hz, and the frequency mismatch between the driving and detection modes is only 1.338 Hz [25].

### 4.6. Surface/Bulk Acoustic Wave Resonators

**Figure 10:**
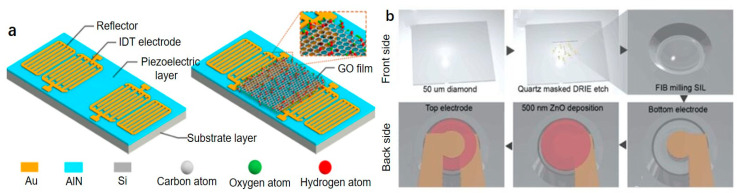
SAW/BAW resonator. (**a**) Aluminum nitride surface acoustic wave humidity sensor with GO film. Adapted with permission from [105]. (**b**) Schematic diagram of the front and back side manufacturing of the SCHBAR. Adapted with permission from [110].

Surface acoustic wave (SAW) refers to the sound wave propagating on and near the solid surface, including Rayleigh wave, Shear horizontal acoustic plate mode, Lamb wave, Love wave. SAW resonators are often used as radiofrequency or intermediate frequency filters. At the same time, due to the transmission characteristics of SAW of the device, they are also widely used in the field of sensors. An SAW sensor has the advantages of small size, high sensitivity, simple processing, and high test accuracy due to its quasi-digital output signal. AlN has the advantages of high phase velocity, high thermal conductivity, and low acoustic loss, which makes it an ideal material for acoustic transmission and is widely used in resonator manufacturing [103] and improved process conditions can control the residual stress of the AlN film to achieve frequency control [104]. Traditional humidity sensors are based on optics, resistance, and capacitance, but the accuracy is low or difficult to integrate. However, surface acoustic wave sensors have the advantages of small size, high sensitivity, and high accuracy. Graphene oxide (GO) has a large surface volume ratio of carbon nanomaterials and has good hydrophilicity and electrical insulation. Le et al. proposed a SAW resonator as shown in Figure 10a, including Si substrate, 1 μm AlN film, 200 nm patterned gold layer as interdigital transducers and reflectors. GO films are deposited on the resonator surface as humidity sensing materials. The transmission spectrum measured the resonant frequency of SAW resonators is about 226.3 MHz. The GO film mass increases nonlinearly in different humidity environments, and results in frequency change is also nonlinear. The absolute sensitivity and relative sensitivity of the resonator reach 125.3 kHz/%RH and 111.7 p.p.m./%RH, respectively [105]. Gao et al. designed AlN S0 lamb wave resonators. The suspended AlN film is sandwiched between the two electrodes, and the bottom electrode is composed of 100 nm Pt. The four corners of the resonator have four tethers to improve the robustness and power-handling of the structure. Lamb wave propagates in the 1 μm AlN film as a plate wave. The designed resonant filter achieves a sizeable fractional bandwidth of 5.6% and a low insertion loss of 1.84 dB. This bandwidth widening capability will have great potential in the 5G new radio field [103]. 

In order to achieve compact and power-efficient sensing applications, Hui et al. designed an ultra-thin piezoelectric plasmonic metasurface, which combines plasmonic resonance and piezoelectric mechanical resonance to achieve high-performance infrared detection. The resonator structure includes a 500 nm thick AlN piezoelectric layer, a 100 nm thick comb-shaped Pt on the bottom layer to drive and detect the lateral mode of the resonator, and a 50 nm gold floating layer on the top to limit electric field penetration. When an AC signal is applied to the driving electrode, if its resonance frequency matches the natural frequency of the resonator, high-order contour-extensional vibration occurs. The metasurface on the top selectively absorbs infrared light to cause the temperature of the resonator to increase, thereby changing the resonance frequency. An uncooled infrared detector with an optimized spectral bandwidth of about 8.8 μm with 80% absorption is realized [106].

The bulk acoustic wave (BAW) resonator uses the inverse piezoelectric effect. Under the action of RF voltage, the piezoelectric layer deforms to generate acoustic waves inside the piezoelectric body. BAW resonators can be used to realize axis-symmetric model matching gyroscopes, achieving megahertz operating frequency at high-quality factors. Due to its high-frequency characteristics, the BAW gyroscope has good stability and strong shock resistance. Serrano et al. fabricated a monocrystalline silicon substrate-decoupled bulk-acoustic wave(SD-BAW) gyroscope using high-aspect-ratio poly- and single-crystal silicon process flow [107]. In BAW resonators, anchor loss dramatically influences the quality factor compared with the squeeze-film damping and the thermoelastic damping. By substrate-decoupled, a disc bulk acoustic resonator with a minimum capacitance gap of 270 nm is fabricated on (100) substrate to reduce anchor loss and achieve a quality factor of up to 60,000. Adjusting the mode’s tuning voltage with a lower resonant frequency can reduce the frequency mismatch from 110 Hz to 0 Hz. The system-level test results show that the angel-random walk coefficient is 0.39°/√h, the bias instability is $10.5{}^{\circ}h^{-1}$ [107].

BAW resonators have various applications. Its electrical properties at gigahertz frequencies can achieve quantum circuit integration and direct quantum control of embedded defect qubits [108]. Compared with nonlinear optics, BAW resonators can also be used to achieve nonlinear acoustics to generate higher harmonics [109]. Farrukh et al. invented a four-port piezoelectric resonator to generate higher harmonic frequency. The top molybdenum layer comprises four interdigitated transducers connected to the resonator’s four ports to provide RF input or output signals. When high-intensity electric fields are applied to the piezoelectric layer, due to the non-centrosymmetric characteristics of AlN piezoelectric material, the nonlinear polarization field leads to high-order harmonic in piezoelectric film. For a single input scanning signal, the resonator obtains two peaks, 106.69 MHz and 121.3 MHz, respectively. While the two input ports employ the same but 180° phase difference signal, the resonance response is considerably improved by common-mode rejection. The three-wave mixing method generates the second harmonic: input specific resonant frequency to two ports and the output signal at the other port. As nonlinear acoustic systems, they can produce high-order harmonics. Resonators with a fundamental frequency of 100 MHz can even produce a frequency band output of 1–10 GHz, which may even exceed nonlinear optical systems [109].

Chen et al. designed a new type of BAW equipment, semiconfocal high-overtone bulk acoustic resonator (SCHBAR), using diamond and SiC materials. BAW resonator has a small mode volume and a high quality factor, which can better realize the single electron-phonon coupling, thereby constructing a strong coupling defect spin-phonon system. As diamond and SiC has excellent mechanical properties and has quantum defects in the crystal lattice, it can be used as the substrate of the resonator, and the thickness of the microresonator is 10–20 μm. The designed resonator is milled into a curved surface by a focused Ga ion beam on one side of the substrate, and a 500 nm thick ZnO is used as a piezoelectric film on the other side to form a sandwich structure with the upper and lower electrodes as shown in Figure 10b. The designed curved structure not only has a higher quality factor but also serves as a solid immersion lens (SIL) and can limit the characteristic dimension of the phonon lateral mode to 10 μm. The diamond substrate has spins from the NV center, which can be addressed optically (laser), magnetically (microwave), and mechanically (acoustics). The SCHBAR device can achieve 3 GHz resonance mode, 20 μm thick diamond device can achieve$\;f\times Q>10^{20}$ at room temperature, and has high strain-driving efficiency, which can be used for fast spin control [110].

### 4.7. Fork Resonators

**Figure 11:**
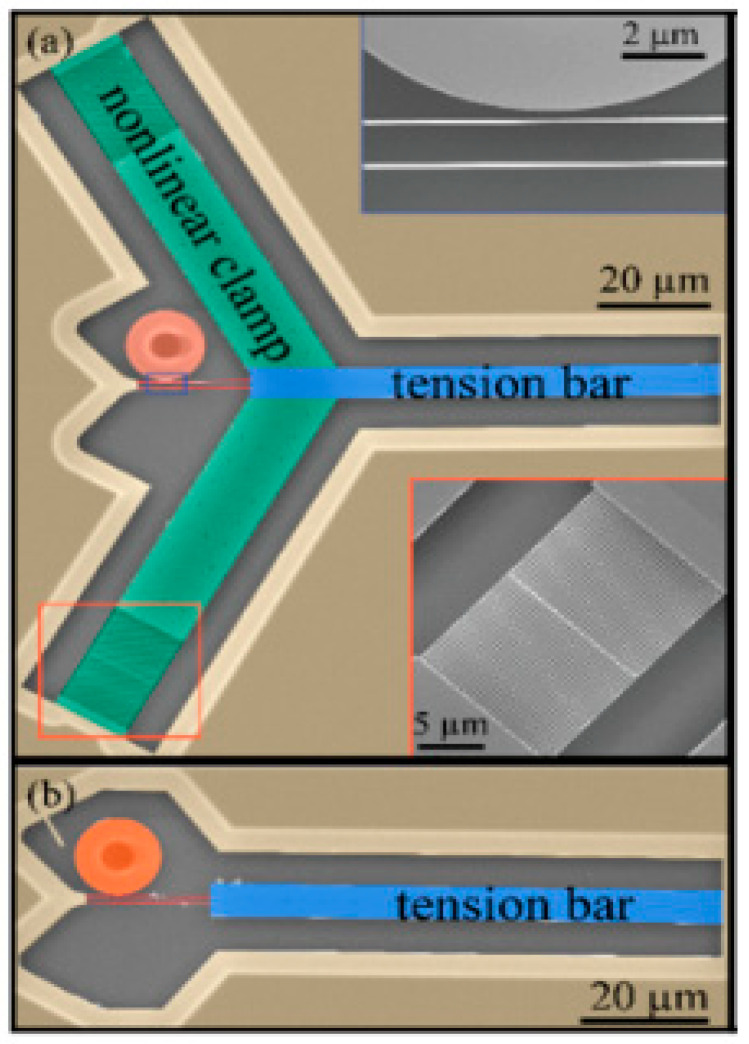
Tuning forks with temperature compensation (**a**) and without temperature compensation (**b**), top inset: coupling of optical readout resonator with tuning fork nanobeam, bottom inset: straightened nonlinear springs. Adapted with permission from [116].

Tuning fork refers to a resonator with two tines. The main application of tuning fork resonators is quartz tuning forks (QTFs) as self-sensing probes [111], which are excited by two electrodes to produce anti-phase oscillation [15]. Due to high stability and high-quality factor, QTFs can be used as mechanical sensors in magnetic force microscope [112], biological sensor [113], and microbalance [114]. With the development of tuning fork resonators, in order to be compatible with mature processing technology in the MEMS field, Si material or AlN material can be used as the tuning fork structure. Lavrik et al. proposed a tuning fork silicon resonator with a coupled beam structure [115]. The resonator includes a pair of coupled resonant beams (tines) that can vibrate in two planes orthogonal to each other. When the two resonant beams are driven to oscillate in antiphase along the drive axis, under the Coriolis force’s action, a differential oscillating output can be achieved in a direction perpendicular to the driving direction. After a trade-off between the resonator’s performance and scale, the simulation shows that the total mass is $2.45\times 10^{-6}\;\mathrm{kg}$ and the resonance frequency is close to 3 kHz. Experiments verify that the excitation frequency is 3.2 kHz in the antiphase mode, which can make each tine move in the opposite direction, the sense frequency response is about 2.67 kHz, and the quality factor is $6.8\times 10^{3}$. “Optical levers” similar to those in atomic force microscopes can achieve differential readout, significantly improve the actual operating performance of the gyroscope, minimize system drifts and noise, and finally get the Allen deviation less than $0.5{}^{\circ}{\;h}^{-1}$ for a time of 1000 s [115]. Wang et al. proposed a tuning fork nanomechanical resonator with high quality factor and high frequency made of silicon nitride materials [116]. Compared with the traditional tuning fork resonator, it has two inclined Y-shaped clamps forming 120° with the bar as shown in Figure 11 [116]. The resonator is composed of two parallel cantilever beams, which are clamped by the long bar on the right side. The nonlinear spring is connected to the support structure. The tuning fork beam, with a length and width of 20 μm and 150 nm respectively, is made of a 250 nm silicon nitride layer grown on silicon dioxide. Each tuning fork is near-field coupled to the silicon nitride disc optical cavity through a gap of about 150 nm to achieve frequency measurement. Tuning fork resonators can achieve relatively constant stress (exceeding the residual stress of the film) by the Y-shaped clamp, so that the thermal expansion coefficient difference between the materials and the temperature change has a small effect on the device stress, achieving f=16.51 MHz, and the frequency sensitivity to temperature as low as −42 Hz/K±14 Hz/K [116].

### 4.8. Other Resonant Structures

Shnaiderman et al. used SOI technology to design a point-like silicon waveguide-etalon detector (SWED) with a sensing area of only 220 nm × 500 nm, which solves the resolution limit of the ultrasonic detector due to its size. The structure of SWED contains a single continuous silicon waveguide, which is composed of four parts: silver layer, spacer, cavity, and Bragg grating. Four SOI chips with different spacer lengths were fabricated. Each chip consists of eight SWEDs, which are arranged adjacent to each other with a spacing of 10 μm. In order to detect ultrasound waves, a continuous-wave laser pumps light into the SWED cavity. To improve the sensitivity, the laser is tuned to be off-resonance and the polarization is maintained in a transverse electrical orientation through a polarization maintaining fiber. The sensitivity per unit area of the SOI based optical resonator is 1000 times higher than that of the microring resonator and 100,000,000 times higher than that of the piezoelectric detector, and it also supports ultrawide detection bandwidth. Moreover, this ultra-miniaturization detector can perform ultrasound imaging with a resolution comparable to that of an optical microscope [117].

The comb drive structure uses capacitors as comb electrodes to generate a high-efficiency electrostatic drive. This structure is often used to drive the proof mass in MEMS accelerometers. The novel gravimeter can also use the capacitive comb electrode to drive the proof mass. Mansouri et al. designed and manufactured an open-loop inertial sensor based on MEMS and ASIC technology, realizing monolithic integration. Resonators structure includes a mass, four slender beams, and four capacitor comb electrodes. This geometric design and large deformation can achieve a stiffness close to zero. Mass movements in the vertical direction are consistent with the direction of gravity, and the transverse comb is driven to reduce the damping caused by the air around the structure [118].

**Figure 12:**
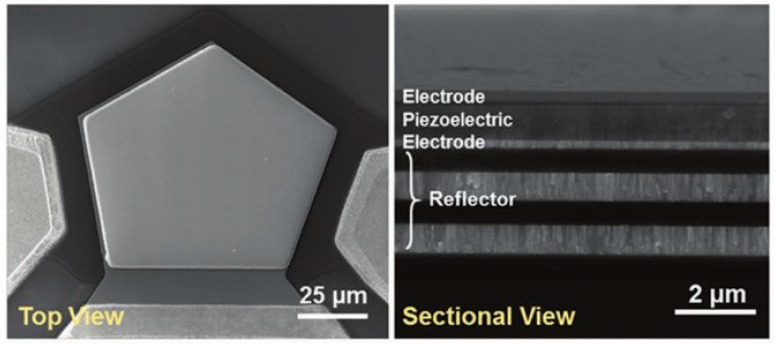
The top and the sectional view of the SEM images of the polygonal hypersonic resonator. Adapted with permission from [119].

Polygonal resonators can focus more energy and suppress other modes, thereby achieving efficient use of acoustic energy. Zhang et al. designed a polygonal resonator as shown in Figure 12 composed of nano-scale piezoelectric layers that can generate gigahertz hypersound and directly interact with the cell membrane to deform and perforate the cell membrane. The piezoelectric layer of the resonator is sandwiched between two metal electrodes to produce a longitudinal mode. The resonance frequency of the resonator is 1.6 GHz. It is placed on the top of the polydimethylsiloxane (PDMS) chamber and aligned with the center of the chamber. Cells are seeded at the bottom of the chamber, and the PDMS chamber is filled with the target drug solution. Due to the pressure of 8.95 MPa generated by hypersound on the cell surface, which is about 59 times that of ultrasound, the interaction between the cell membrane and hypersound produces normal stress and shear stress, resulting in the generation of instantaneous nanopores, which significantly promotes the uptake of target drugs by cells [119].

## 5. Conclusions

As discussed in this article, MEMS/NEMS resonators can be designed as single-ended/double-ended clamped structures, hemispherical shells, microdisk, drum, ring, fork, SAW/BAW, and other original resonant structure. The materials, dimensions, resonance frequency, quality factor, excitation/detection methods, and applications of different resonators’ structures are summarized in Table 1. The resonance applications of the same structure in Table 1 are sorted according to the resonance frequency from small to large. Resonators can use piezoelectric, electrical, and optical excitation, using non-destructive acoustic testing, magnetomotive transduction testing, and laser testing. Some new processes that fabricate resonators such as VLS epitaxial growth can produce nanowire resonators with higher crystal quality and surface properties, and FEBID can achieve large aspect ratios and in-situ growth capabilities. The frequency and mode of the resonator are important characteristics of resonance. Tune the resonant mode by controlling the gate voltage of the source and drain, or use the pin junction to adjust the width of the depletion region to tune the resonant frequency. Single/double-clamped resonators can be used as MTA to analyze the phase change process according to the change of the material resonance frequency during the thermal cycle. In the quantum field, resonators can be used to analyze the impact of quantum mechanics on macroscopic mechanical oscillations or magnetically couple with atoms. The resonator can also be used as mass sensors to measure molecular mass in the MDa range or as an oscillator to realize sustained oscillation.

The hemispherical shell and ring structure resonator have excellent performance as gyroscopes. The resonance frequency is in the kHz range. The hemispherical resonant structure is usually optically tested, while the ring structure gyroscope is driven by electrostatic. In order to reduce loss, gyroscopes have extremely high quality factors, which can reach 510,000. Microdisk resonators can work at megahertz or gigahertz, and as high-frequency resonators, optical detection can be used to study the resonant multi-mode or energy loss. The drum resonator is made of thin-film materials. For example, a graphene drum resonator has various applications such as a high-frequency random switch, oscillator, or octave frequency tuning. SAW/BAW resonators are mainly manufactured by the inverse piezoelectric effect. AlN materials are often used as piezoelectric layers to work in the megahertz range. When the GO film material is deposited on the surface of the piezoelectric layers, it can be used as a humidity sensor. When the gold floating layer is deposited, it can form a metasurface to realize infrared detection.

The resonator with multiple resonant structures still has exploratory value in many fields in the future. As sensors, it can study nonlinear sensing applications and the influence of non-specific adsorption. As gyroscopes, the main goal is to reduce energy loss, improve quality factors, and achieve better mode matching and stability. However, as filter devices and oscillators, it is necessary to realize better the filtering of noise and the rapid switching of frequencies. In the quantum field, the coupling of nanoresonators and spin states or the influence of quantum mechanical fluctuations on resonance can be studied.

## Figures and Tables

**Table 1:** Resonance performance, application, and excitation/detection methods of resonators with different structures.

Structure	Material	Dimension	Frequency	Quality Factor	Excitation/Detection	Application	Reference
**Double-clamped**	Carbon nanotubes	1005.9 μm (l)	500 Hz		acoustic drive	Anti-fatigue property	Ref [35]
	${\mathrm{VO}}_{2}$	100 (l)×5(w)μm	32.6 kHz		electric drive	Self oscillation	Ref [92]
	Pyrolytic carbon	400 (l)×30 (w)×0.6 (t) µm	233 ± 4 KHz		electric drive/detection	MTA	Ref [65]
	Si		352.2 KHz	30,160	differential drive	Accelerometer	Ref [120]
	Si	800 (l)×280 (w) nm	1.26 MHz		electric drive	Self oscillation	Ref [91]
	Carbon nanotubes	4 μm (l)	4.2 MHz	48,000	electrostatic drive /mixing detection	Force sensing	Ref [56]
	h-BN	6.7 nm (*t*)	14.06 MHz	39	thermodynamic fluctuation	Resonator	Ref [121]
	Carbon nanotubes	1 μm (l)	46–51.5 MHz		electric drive/detection	Energy loss	Ref [59]
	Si	7.7 (l)×0.33 (w)×0.8 (t) μm	70.72 MHz	$1.8\times 10^{4}$	magnetomotive transduction	High frequency Resonator	Ref [36]
	Single-layer graphene	1.1 (l)×1.93 (w)×0.0003 (t) μm	70.5 MHz	78	optical drive	Resonator	Ref [122]
	Silicon nanowire	1.8 (l)×0.04 (t) μm	96 MHz	5500	electric drive	Electromechanical system	Ref [40]
	GaAs	3 (l)×0.25 (w)×0.2 (t) μm	116.7 MHz	1700	SET detection	Quantum mechanics	Ref [70]
	Silicon nitride	2 (l)×0.165 (w)×0.125 (t) μm	145 MHz	400	laser drive/detection	Energy loss	Ref [33]
	SiC	2.3 (l)×0.15 (w)×0.1 (t) μm	190 MHz	5000	reflection bridge	Mass sensing	Ref [82]
	Silicon nanowire	2.25 (l)×0.142 (w) μm	200 MHz	2000	magnetomotive transduction	High frequency resonator	Ref [32]
**Single-clamped**	${\mathrm{Fe}}_{0.7}{\mathrm{Ga}}_{0.3}$/PZT	1000 (l)×200 (w)×5 (t) μm	3.549 KHz	2500	electric/ magnetic drive	Resonance tuning	Ref [39]
	TP MH	1000 (l)×65 (w) μm	19±3.8 kHz		piezoelectric excitation	MTA	Ref [67]
	Carbon nanotubes	1–15 (l) μm	57.04 kHz	3000	optomechanical detection	Mass sensing	Ref [34]
	Pt-C	5 (l) μm	662.5 kHz.	149±13	electrical detection	Original process	Ref [45]
	SiC nanowire	10 (l)×0.05 (d) μm	1 MHz	10,000	optical detection	Spin coupling	Ref [79]
	Si	7 (l)×0.2 (w)×0.1 (t) μm	1.12 MHz	5000		Atomic spin	Ref [21]
	GaAs	4 (l)×0.8 (w)×0.2 (t) μm	8 MHz	2700	piezoelectric excitation	Resonance tuning	Ref [47]
	Porous nano cantilever	4 (l)×0.130 (w)×0.220 (t) μm	9.66 MHz			Mass sensing	Ref [88]
	Si	5 (l)×0.3 μm	19.16 MHz	5000	electrical detection	Mass sensing	Ref [85,86]
	Si	5–10 (l) μm	20–120 MHz	7500–8500	electrical detection	Mass sensing	Ref [87]
	SiC	0.6 (l)×0.4 (w)×0.1(t) μm	127 MHz	900	electrical detection	High frequency resonator	Ref [1]
	Carbon nanotubes	205 (l)×1.78 (d) nm	328.5 MHz	1000	radio signal detect	Mass sensing	Ref [81]
**Hemispherical Shell**	Metallic glasses	3 mm (*d*)	13.944 kHz	6200	optical detection	Gyroscope	Ref [13]
	Polycrystalline diamond	1.1 mm (*d*)	18.316 kHz	20,000	optical detection	Resonator	Ref [93]
**Ring**	Si	1–6 mm (*r*)	2163.8 Hz	510,000	electrostatic drive	Gyroscope	Ref [25]
	Si	720 μm (*d*)	134.31 kHz		electrostatic drive	Resonator	Ref [27]
**Microdisk**	Diamond	38 μm (*d*)	9–30 MHz	>$10^{4}$	laser detection	Mass sensing	Ref [24]
	SiC	19.5 μm (*d*)	10.14~16.48 MHz	850~1360	laser detection	Multi-mode	Ref [14]
	GaAs	1–3 μm (*r*)	1.3 GHz		optomechanical detection	Energy loss	Ref [97]
**Drum**	SiN	100 nm (*t*)	121.1 kHz	$5.3\times 10^{6}$	optical interference	Octave frequency tuning	Ref [23]
	Graphene	285 nm (*t*)	13.92 MHz	416.6		High-frequency stochastic switch	Ref [26]
	Graphene	4 μm (*d*)	52.19 MHz	55	electric drive	Oscillator	Ref [99]
	Graphene	4 μm (*d*)	60–75 MHz	500–3000	optical detection	In plane stress detection	Ref [100]
**Fork**	Si		3 KHz	$6.8\times 10^{3}$	optical levers detection	Resonator	Ref [115]
	Silicon nitride	20 μm(l)	16.51 MHz			Resonator	Ref [116]
**SAW/BAW**	Si		4.3 MHz	60,000	electrostatic excitation	Gyroscope	Ref [107]
	AlN	1 μm (*t*)	106.69 MHz		electrical excitation	High-order harmonics	Ref [109]
	AlN/Au	500 nm (*t*)	161.4 MHz	1116	electrical excitation	Infrared detector	Ref [106]
	Graphene oxide/AlN	1 μm (*t*)	226.3 MHz		transmission spectrum detection	Humidity sensor	Ref [105]
	AlN	1 μm (*t*)	446 MHz	1500	RF excitation	Resonator	Ref [103]
	Diamond/ZnO	10–20 μm (*t*)	3 GHz			Fast spin control	Ref [110]

## References

[1] Li M., Tang H.X., Roukes M.L. (2007). Ultra-sensitive NEMS-based cantilevers for sensing, scanned probe and very high-frequency applications. Nat. Nanotechnol..

[2] Johnson B.N., Mutharasan R. (2012). Biosensing using dynamic-mode cantilever sensors: A review. Biosens. Bioelectron..

[3] Arash B., Jiang J.-W., Rabczuk T. (2015). A review on nanomechanical resonators and their applications in sensors and molecular transportation. Appl. Phys. Rev..

[4] Gouttenoire V., Barois T., Perisanu S., Leclercq J.-L., Purcell S.T., Vincent P., Ayari A. (2010). Digital and FM demodulation of a doubly clamped single-walled carbon-nanotube oscillator: Towards a nanotube cell phone. Small.

[5] Jose-Yacaman M., Miki-Yoshida M., Rendon L., Santiesteban J.G. (1993). Catalytic growth of carbon microtubules with fullerene structure. Appl. Phys. Lett..

[6] Li S., Feng X., Liu H., Wang K., Long Y.-Z., Ramakrishna S. (2019). Preparation and application of carbon nanotubes flexible sensors. J. Semicond..

[7] Wilder J.W.G., Venema L.C., Rinzler A.G., Smalley R.E., Dekker C. (1998). Electronic structure of atomically resolved carbon nanotubes. Nat. Cell Biol..

[8] Yu C., Liu Q., He Z., Gao X., Wu E., Guo J., Zhou C., Feng Z. (2020). Epitaxial graphene gas sensors on SiC substrate with high sensitivity. J. Semicond..

[9] Huang M.H., Wu Y., Feick H., Tran N., Weber E., Yang P. (2001). Catalytic growth of zinc oxide nanowires by vapor transport. Adv. Mater..

[10] Samanta C., Gangavarapu P.R.Y., Naik A.K. (2015). Nonlinear mode coupling and internal resonances in MoS2 nanoelectromechanical system. Appl. Phys. Lett..

[11] Mehmood Z., Haneef I., Udrea F. (2018). Material selection for Micro-Electro-Mechanical-Systems (MEMS) using Ashby’s approach. Mater. Des..

[12] Middlemiss R.P., Samarelli A., Paul D.J., Hough J., Rowan S., Hammond G.D. (2016). Measurement of the Earth tides with a MEMS gravimeter. Nat. Cell Biol..

[13] Kanik M., Bordeenithikasem P., Kim D., Selden N., Desai A., M’Closkey R., Schroers J. (2014). Metallic glass hemispherical shell resonators. J. Microelectromechanical Syst..

[14] Wang Z., Lee J., Feng P. (2014). Spatial mapping of multimode Brownian motions in high-frequency silicon carbide microdisk resonators. Nat. Commun..

[15] Abrahamians J.-O., Van L.P., Régnier S. (2016). Contributed review: Quartz force sensing probes for micro-applications. Rev. Sci. Instrum..

[16] Castelletto S., Rosa L., Blackledge J., Al Abri M.Z., Boretti A. (2017). Advances in diamond nanofabrication for ultrasensitive devices. Microsyst. Nanoeng..

[17] Fritz J., Baller M.K., Lang H.P., Rothuizen H., Vettiger P., Meyer E., Güntherodt H.-J., Gerber C., Gimzewski J.K. (2000). Translating biomolecular recognition into nanomechanics. Science.

[18] Song P., Ma Z., Ma J., Yang L., Wei J., Zhao Y., Zhang M., Yang F., Wang X. (2020). Recent progress of miniature MEMS pressure sensors. Micromachines.

[19] Campbell G.A., Uknalis J., Tu S.-I., Mutharasan R. (2007). Detect of Escherichia coli O157:H7 in ground beef samples using piezoelectric excited millimeter-sized cantilever (PEMC) sensors. Biosens. Bioelectron..

[20] Wallraff A., Schuster D.I., Blais A., Frunzio L., Huang R.-S., Majer J., Kumar S., Girvin S.M., Schoelkopf R.J. (2004). Strong coupling of a single photon to a superconducting qubit using circuit quantum electrodynamics. Nat. Cell Biol..

[21] Treutlein P., Hunger D., Camerer S., Hänsch T.W., Reichel J. (2007). Bose-Einstein condensate coupled to a nanomechanical resonator on an atom chip. Phys. Rev. Lett..

[22] Stripling W., Baskett J.R. (1992). Hemispherical Resonator Gyro: Principle, Design, and Performance.

[23] St-Gelais R., Bernard S., Reinhardt C., Sankey J.C. (2019). Swept-frequency drumhead optomechanical resonators. ACS Photonics.

[24] Sartori A.F., Belardinelli P., Dolleman R., Steeneken P.G., Ghatkesar M.K., Buijnsters J.G. (2019). Inkjet-printed high-Q nanocrystalline diamond resonators. Small.

[25] Li Q., Xiao D., Zhou X., Xu Y., Zhuo M., Hou Z., He K., Zhang Y., Wu X. (2018). 0.04 degree-per-hour MEMS disk resonator gyroscope with high-quality factor (510 k) and long decaying time constant (74.9 s). Microsyst. Nanoeng..

[26] Dolleman R.J., Belardinelli P., Houri S., Van Der Zant H.S.J., Alijani F., Steeneken P.G. (2019). High-Frequency Stochastic Switching of Graphene Resonators Near Room Temperature. Nano Lett..

[27] Zhou X., Zhao C., Xiao D., Sun J., Sobreviela G., Gerrard D.D., Chen Y., Flader I., Kenny T.W., Wu X. (2019). Dynamic modulation of modal coupling in microelectromechanical gyroscopic ring resonators. Nat. Commun..

[28] Wu T.-T., Chen Y.-Y., Chou T.-H. (2008). A high sensitivity nanomaterial based SAW humidity sensor. J. Phys. D Appl. Phys..

[29] Ferrier D.C., Shaver M.P., Hands P.J. (2015). Micro- and nano-structure based oligonucleotide sensors. Biosens. Bioelectron..

[30] Cooper M.A., Singleton V.T. (2007). A survey of the 2001 to 2005 quartz crystal microbalance biosensor literature: Applications of acoustic physics to the analysis of biomolecular interactions. J. Mol. Recognit..

[31] Khudiyev T., Clayton J.D., Levy E.C., Chocat N., Gumennik A., Stolyarov A.M., Joannopoulos J., Fink Y. (2017). Electrostrictive microelectromechanical fibres and textiles. Nat. Commun..

[32] Feng X., He R., Yang P., Roukes M.J.N.L. (2007). Very high frequency silicon nanowire electromechanical resonators. Nano Lett..

[33] Verbridge S.S., Bellan L.M., Parpia J.M., Craighead H.G. (2006). Optically driven resonance of nanoscale flexural oscillators in liquid. Nano Lett..

[34] Gruber G., Urgell C., Tavernarakis A., Stavrinadis A., Tepsic S., Magen C., Sangiao S., De Teresa J.M., Verlot P., Bachtold A. (2019). Mass sensing for the advanced fabrication of nanomechanical resonators. Nano Lett..

[35] Bai Y., Yue H., Wang J., Shen B., Sun S., Wang S., Wang H., Li X., Xu Z., Zhang R. (2020). Super-durable ultralong carbon nanotubes. Science.

[36] Cleland A.N., Roukes M.L. (1996). Fabrication of high frequency nanometer scale mechanical resonators from bulk Si crystals. Appl. Phys. Lett..

[37] Wang D., Zhang Z., Li B., Duan X. (2021). Synthesis of two-dimensional/one-dimensional heterostructures with tunable width. J. Semicond..

[38] Liao M., Sang L., Teraji T., Koizumi S., Koide Y. (2019). Ultrahigh performance on-chip single crystal diamond NEMS/MEMS with electrically tailored self-sensing enhancing actuation. Adv. Mater. Technol..

[39] Onuta T.-D., Wang Y., Lofland S.E., Takeuchi I. (2014). Multiferroic Operation of dynamic memory based on heterostructured cantilevers. Adv. Mater..

[40] He R., Feng P., Roukes M.L., Yang P. (2008). Self-transducing silicon nanowire electromechanical systems at room temperature. Nano Lett..

[41] Kumar S., Bhushan P., Pandey M., Bhattacharya S. (2019). Additive manufacturing as an emerging technology for fabrication of microelectromechanical systems (MEMS). J. Micromanuf..

[42] Credi C., Fiorese A., Tironi M., Bernasconi R., Magagnin L., Levi M., Turri S. (2016). 3D printing of cantilever-type microstructures by stereolithography of ferromagnetic photopolymers. ACS Appl. Mater. Interfaces.

[43] Leary J.F., Key J., Vidi P.-A., Cooper C.L., Kole A., Reece L.M., Lelièvre S.A. Human organ-on-a-chip BioMEMS devices for testing new diagnostic and therapeutic strategies. Proceedings of the Microfluidics, BioMEMS, and Medical Microsystems XI Conference.

[44] Teh K.S. (2017). Additive direct-write microfabrication for MEMS: A review. Front. Mech. Eng..

[45] Arnold G., Winkler R., Stermitz M., Orthacker A., Noh J., Fowlkes J.D., Kothleitner G., Huth M., Rack P.D., Plank H. (2018). Tunable 3D nanoresonators for gas-sensing applications. Adv. Funct. Mater..

[46] Song P., Si C., Zhang M., Zhao Y., He Y., Liu W., Wang X. (2020). A novel piezoresistive MEMS pressure sensors based on temporary bonding technology. Sensors.

[47] 4Masmanidis S.C., Karabalin R.B., De Vlaminck I., Borghs G., Freeman M.R., Roukes M.L. (2007). Multifunctional nanomechanical systems via tunably coupled piezoelectric actuation. Science.

[48] Schmid S., Villanuevam L.G., Roukes M.L. (2016). Fundamentals of Nanomechanical Resonators.

[49] Huang X.M.H., Zorman C.A., Mehregany M., Roukes M.L. (2003). Nanodevice motion at microwave frequencies. Nat. Cell Biol..

[50] Zhou J., Moldovan N., Stan L., Cai H., Czaplewski D.A., López D. (2020). Approaching the strain-free limit in ultrathin nanomechanical resonators. Nano Lett..

[51] Roy S.K., Sauer V.T.K., Westwood-Bachman J.N., Venkatasubramanian A., Hiebert W.K. (2018). Improving mechanical sensor performance through larger damping. Science.

[52] Tajaddodianfar F., Yazdi M.R.H., Pishkenari H.N. (2016). Nonlinear dynamics of MEMS/NEMS resonators: Analytical solution by the homotopy analysis method. Microsyst. Technol..

[53] Jin L., Zhao H., Li Z., Jiang Z., Li L., Yan X. (2021). Nonlinear dynamic control of GaAs nanomechanical resonators using lasers. Nanotechnology.

[54] Maillet O., Zhou X., Gazizulin R., Cid A.M., Defoort M., Bourgeois O., Collin E. (2017). Nonlinear frequency transduction of nanomechanical Brownian motion. Phys. Rev. B.

[55] Barnard A.W., Zhang M., Wiederhecker G.S., Lipson M., McEuen P.L. (2019). Real-time vibrations of a carbon nanotube. Nat. Cell Biol..

[56] Moser J., Güttinger J., Eichler A., Esplandiu M.J., Liu D.E., Dykman M.I., Bachtold A. (2013). Ultrasensitive force detection with a nanotube mechanical resonator. Nat. Nanotechnol..

[57] Guzman P., Dinh T., Phan H.-P., Joy A.P., Qamar A., Bahreyni B., Zhu Y., Rais-Zadeh M., Li H., Nguyen N.-T. (2020). Highly-doped SiC resonator with ultra-large tuning frequency range by Joule heating effect. Mater. Des..

[58] Jia H., Feng P.X.-L. (2019). Very high-frequency silicon carbide microdisk resonators with multimode responses in water for particle sensing. J. Microelectromechanical Syst..

[59] Aykol M., Hou B., Dhall R., Chang S.-W., Branham W., Qiu J., Cronin S.B. (2014). Clamping instability and Van der Waals forces in carbon nanotube mechanical resonators. Nano Lett..

[60] Ghadimi A.H., Fedorov S.A., Engelsen N.J., Bereyhi M.J., Schilling R., Wilson D.J., Kippenberg T.J. (2018). Elastic strain engineering for ultralow mechanical dissipation. Science.

[61] Bao F.-H., Bao J.-F., Lee J.E.-Y., Bao L.-L., Khan M.A., Zhou X., Wu Q.-D., Zhang T., Zhang X.-S. (2019). Quality factor improvement of piezoelectric MEMS resonator by the conjunction of frame structure and phononic crystals. Sens. Actuators A Phys..

[62] Miao T., Hu X., Zhou X., Wu X., Hou Z., Xiao D. A million-order effective quality factor MEMS resonator by mechanical pumping. Proceedings of the 2020 IEEE International Symposium on Inertial Sensors and Systems (INERTIAL).

[63] Wunderlich B.J.J. (2011). Methodology of interpreting thermal analysis of polymers. J. Therm. Anal. Calorim..

[64] Magoshi J., Becker M.A., Han Z., Nakamura S. (2002). Thermal properties of seed proteins. J. Therm. Anal. Calorim..

[65] Nguyen L.Q., Larsen P.E., Larsen T., Goswami S.B., Villanueva L.G., Boisen A., Keller S.S. (2019). Pyrolytic carbon resonators for micromechanical thermal analysis. Microsyst. Nanoeng..

[66] Jung N., Jeon S. (2008). Nanomechanical thermal analysis with silicon cantilevers of the mechanical properties of poly(vinyl acetate) near the glass transition temperature. Macromolecules.

[67] Okeyo P.O., Larsen P.E., Kissi E.O., Ajalloueian F., Rades T., Rantanen J., Boisen A. (2020). Single particles as resonators for thermomechanical analysis. Nat. Commun..

[68] Van Frank S., Bonneau M., Schmiedmayer J., Hild S., Gross C., Cheneau M., Bloch I., Pichler T., Negretti A., Calarco T. (2016). Optimal control of complex atomic quantum systems. Sci. Rep..

[69] Cho A. (2003). Physics: Researchers Race to Put the Quantum into Mechanics. Science.

[70] Knobel R.G., Cleland A.N. (2003). Nanometre-scale displacement sensing using a single electron transistor. Nat. Cell Biol..

[71] Kalaee M., Mirhosseini M., Dieterle P.B., Peruzzo M., Fink J.M., Painter O. (2019). Quantum electromechanics of a hypersonic crystal. Nat. Nanotechnol..

[72] Chan J., Safavi-Naeini A.H., Hill J., Meenehan S., Painter O. (2012). Optimized optomechanical crystal cavity with acoustic radiation shield. Appl. Phys. Lett..

[73] MacCabe G.S., Ren H., Luo J., Cohen J.D., Zhou H., Sipahigil A., Mirhosseini M., Painter O. (2020). Nano-acoustic resonator with ultralong phonon lifetime. Science.

[74] Wang H., Lekavicius I. (2020). Coupling spins to nanomechanical resonators: Toward quantum spin-mechanics. Appl. Phys. Lett..

[75] Carter S.G., Bracker A.S., Bryant G.W., Kim M., Kim C.S., Zalalutdinov M.K., Yakes M.K., Czarnocki C., Casara J., Scheibner M. (2018). Spin-mechanical coupling of an InAs quantum dot embedded in a mechanical resonator. Phys. Rev. Lett..

[76] Oeckinghaus T., Momenzadeh S.A., Scheiger P., Shalomayeva T., Finkler A., Dasari D.B.R., Stöhr R., Wrachtrup J. (2019). Spin–phonon interfaces in coupled nanomechanical cantilevers. Nano Lett..

[77] O’Connell A.D., Hofheinz M., Ansmann M., Bialczak R.C., Lenander M., Lucero E., Neeley M., Sank D., Wang H., Weides M. (2010). Quantum ground state and single-phonon control of a mechanical resonator. Nat. Cell Biol..

[78] Blatt R., Wineland D. (2008). Entangled states of trapped atomic ions. Nat. Cell Biol..

[79] Arcizet O., Jacques V., Siria A., Poncharal P., Vincent P., Seidelin S. (2011). A single nitrogen-vacancy defect coupled to a nanomechanical oscillator. Nat. Phys..

[80] Fortágh J., Zimmermann C. (2007). Magnetic microtraps for ultracold atoms. Rev. Mod. Phys..

[81] Jensen K.H., Kim K., Zettl A. (2008). An atomic-resolution nanomechanical mass sensor. Nat. Nanotechnol..

[82] Yang Y.T., Callegari C., Feng P., Ekinci K.L., Roukes M.L., Yang Y.T., Callegari C., Feng P., Ekinci K.L., Roukes M.L. (2006). Zeptogram-scale nanomechanical mass sensing. Nano Lett..

[83] Mujahid A., Dickert F.L. (2017). Surface acoustic wave (SAW) for chemical sensing applications of recognition layers. Sensors.

[84] 3Hanay M.S., Kelber S., Naik A., Chi D., Hentz S., Bullard E.C., Colinet E., Duraffourg L., Roukes M.L. (2012). Single-protein nanomechanical mass spectrometry in real time. Nat. Nanotechnol..

[85] Mile E., Jourdan G., Bargatin I., Labarthe S., Marcoux C., Andreucci P., Hentz S., Kharrat C., Colinet E., Duraffourg L. (2010). In-plane nanoelectromechanical resonators based on silicon nanowire piezoresistive detection. Nanotechnology.

[86] Dominguez-Medina S., Fostner S., Defoort M., Sansa M., Stark A.-K., Halim M.A., Vernhes E., Gely M., Jourdan G., Alava T. (2018). Neutral mass spectrometry of virus capsids above 100 megadaltons with nanomechanical resonators. Science.

[87] Sage E., Sansa M., Fostner S., Defoort M., Gély M., Naik A.K., Morel R., Duraffourg L., Roukes M.L., Alava T. (2018). Single-particle mass spectrometry with arrays of frequency-addressed nanomechanical resonators. Nat. Commun..

[88] Venkatasubramanian A., Sauer V.T.K., Westwood-Bachman J.N., Cui K., Xia M., Wishart D.S., Hiebert W.K. (2019). Porous nanophotonic optomechanical beams for enhanced mass adsorption. ACS Sensors.

[89] Anderson P.W. (1958). Absence of diffusion in certain random lattices. Phys. Rev..

[90] Wang D.F., Du X., Li X., Zhou D., Xia C., Zheng G., Wan S., Wang X. (2018). Picogram-order mass sensors via cantilever-based micro-/nanostructures. Micro Electro Mechanical Systems.

[91] Steeneken P., Phan K.L., Goossens M.J., Koops G.E.J., Brom G.J.A.M., Van Der Avoort C., Van Beek J.T.M. (2011). Piezoresistive heat engine and refrigerator. Nat. Phys..

[92] Manca N., Pellegrino L., Kanki T., Venstra W.J., Mattoni G., Higuchi Y., Tanaka H., Caviglia A.D., Marré D. (2017). Selective high-frequency mechanical actuation driven by the VO2 electronic instability. Adv. Mater..

[93] Heidari A., Chan M.-L., Yang H.-A., Jaramillo G., Taheri-Tehrani P., Fonda P., Najar H., Yamazaki K., Lin L., Horsley D. (2013). Hemispherical wineglass resonators fabricated from the microcrystalline diamond. J. Micromech. Microeng..

[94] Nguyen D.T., Baker C., Hease W., Sejil S., Senellart P., Lemaitre A., Ducci S., Leo G., Favero I. (2013). Ultrahigh Q-frequency product for optomechanical disk resonators with a mechanical shield. Appl. Phys. Lett..

[95] Nguyen D.T., Hease W., Baker C., Gil-Santos E., Senellart P., Lemaître A., Ducci S., Leo G., Favero I. (2015). Improved optomechanical disk resonator sitting on a pedestal mechanical shield. N. J. Phys..

[96] Gil-Santos E., Ruz J.J., Malvar O., Favero I., Lemaître A., Kosaka P.M., García-López S., Calleja M., Tamayo J. (2020). Optomechanical detection of vibration modes of a single bacterium. Nat. Nanotechnol..

[97] Gil Santos E., Baker C., Nguyen D.T., Hease W., Gomez C., Lemaitre A., Ducci S., Leo G., Favero I. (2015). High-frequency nano-optomechanical disk resonators in liquids. Nat. Nanotechnol..

[98] Cai X., Han X., Zhao C., Niu C., Jia Y. (2020). Tellurene: An elemental 2D monolayer material beyond its bulk phases without Van der Waals layered structures. J. Semicond..

[99] Chen C., Lee S., Deshpande V.V., Lee G.-H., Lekas M., Shepard K., Hone J. (2013). Graphene mechanical oscillators with tunable frequency. Nat. Nanotechnol..

[100] Robinson J.T., Zalalutdinov M.K., Cress C.D., Culbertson J.C., Friedman A.L., Merrill A., Landi B.J. (2017). Graphene strained by defects. ACS Nano.

[101] Karg T.M., Gouraud B., Treutlein P., Hammerer K. (2019). Remote Hamiltonian interactions mediated by light. Phys. Rev. A.

[102] Karg T.M., Gouraud B., Ngai C.T., Schmid G.-L., Hammerer K., Treutlein P. (2020). Light-mediated strong coupling between a mechanical oscillator and atomic spins 1 meter apart. Science.

[103] Gao A., Liu K., Liang J., Wu T. (2020). AlN MEMS filters with extremely high bandwidth widening capability. Microsyst. Nanoeng..

[104] Ross G., Dong H., Karuthedath C.B., Sebastian A.T., Pensala T., Paulasto-Kröckel M. (2020). The impact of residual stress on resonating piezoelectric devices. Mater. Des..

[105] Le X., Liu Y., Peng L., Pang J., Xu Z., Gao C., Xie J. (2019). Surface acoustic wave humidity sensors based on uniform and thickness controllable graphene oxide thin films formed by surface tension. Microsyst. Nanoeng..

[106] Hui Y., Gomez-Diaz J.S., Qian Z., Alù A., Rinaldi M. (2016). Plasmonic piezoelectric nanomechanical resonator for spectrally selective infrared sensing. Nat. Commun..

[107] Serrano D.E., Zaman M.F., Rahafrooz A., Hrudey P., Lipka R., Younkin D., Nagpal S., Jafri I., Ayazi F. (2016). Substrate-decoupled, bulk-acoustic wave gyroscopes: Design and evaluation of next-generation environmentally robust devices. Microsyst. Nanoeng..

[108] Chu Y., Kharel P., Renninger W.H., Burkhart L.D., Frunzio L., Rakich P.T., Schoelkopf R.J. (2017). Quantum acoustics with superconducting qubits. Science.

[109] Mateen F., Boales J., Erramilli S., Mohanty P. (2018). Micromechanical resonator with dielectric nonlinearity. Microsyst. Nanoeng..

[110] Chen H., Opondo N.F., Jiang B., MacQuarrie E.R., Daveau R.S., Bhave S.A., Fuchs G.D. (2019). Engineering electron–phonon coupling of quantum defects to a semiconfocal acoustic resonator. Nano Lett..

[111] Karrai K., Grober R.D. (1995). Piezoelectric tip-sample distance control for near field optical microscopes. Appl. Phys. Lett..

[112] Seo Y., Cadden-Zimansky P., Chandrasekhar V. (2005). Low-temperature high-resolution magnetic force microscopy using a quartz tuning fork. Appl. Phys. Lett..

[113] Su X., Dai C., Zhang J., O’Shea S.J. (2002). Quartz tuning fork biosensor. Biosens. Bioelectron..

[114] Nihei F., Ideura K., Kobayashi H., Taniguchi J., Suzuki M. (2010). Mechanical response of 4He films adsorbed on graphite with a quartz tuning fork. J. Low Temp. Phys..

[115] Lavrik N.V., Datskos P.G. (2019). Optically read Coriolis vibratory gyroscope based on a silicon tuning fork. Microsyst. Nanoeng..

[116] Wang M., Zhang R., Ilic R., Aksyuk V., Liu Y. (2020). Frequency stabilization of nanomechanical resonators using thermally invariant strain engineering. Nano Lett..

[117] Shnaiderman R., Wissmeyer G., Ülgen O., Mustafa Q., Chmyrov A., Ntziachristos V. (2020). A submicrometre silicon-on-insulator resonator for ultrasound detection. Nat. Cell Biol..

[118] El Mansouri B., Middelburg L.M., Poelma R., Zhang G.Q., Van Zeijl H.W., Wei J., Jiang H., Vogel J.G., Van Driel W.D. (2019). High-resolution MEMS inertial sensor combining large-displacement buckling behaviour with integrated capacitive readout. Microsyst. Nanoeng..

[119] Zhang Z., Wang Y., Zhang H., Tang Z., Liu W., Lu Y., Wang Z., Yang H., Pang W., Zhang H. (2017). Hypersonic poration: A new versatile cell poration method to enhance cellular uptake using a piezoelectric nano-electromechanical device. Small.

[120] Zhao C., Pandit M., Sobreviela G., Steinmann P., Mustafazade A., Zou X., Seshia A. (2019). A resonant MEMS accelerometer with 56ng bias stability and 98ng/Hz1/2 noise floor. J. Microelectromechanical Syst..

[121] Zheng X.-Q., Lee J., Feng P.X.-L. (2017). Hexagonal boron nitride nanomechanical resonators with spatially visualized motion. Microsyst. Nanoeng..

[122] Bunch J.S., Van der Zande A.M., Verbridge S.S., Frank I.W., Tanenbaum D.M., Parpia J.M., Craighead H.G., McEuen P.L. (2007). Electromechanical resonators from graphene sheets. Science.

